# Cross-Cultural Adaptation of Instruments Measuring Children’s Movement Behaviors and Parenting Practices in Brazilian Families

**DOI:** 10.3390/ijerph18010239

**Published:** 2020-12-31

**Authors:** Widjane Goncalves, Rebecca Byrne, Pedro Lira, Marcelo Viana, Stewart G. Trost

**Affiliations:** 1 Institute of Health and Biomedical Innovation at Queensland Centre for Children’s Health Research, South Brisbane 4101, Australia; widjane.ferreiragoncalves@hdr.qut.edu.au (W.G.); ra.byrne@qut.edu.au (R.B.); 2 School of Exercise and Nutrition Sciences, Queensland University of Technology (QUT), Kelvin Grove 4059, Australia; 3 Department of Nutrition, Federal University of Pernambuco, Recife-Pernambuco 50670-901, Brazil; lirapic@ufpe.br (P.L.); mtviana0@hotmail.com (M.V.)

**Keywords:** translations, cognitive interviewing, physical activity, screen time, sleep, child, parents

## Abstract

Childhood obesity is a global problem, disproportionately affecting children in low-to-middle income countries (LMIC). Despite this evidence, no previous study has adapted instruments measuring children’s movement behaviors and associated parenting practices for use in LMIC families. This study reports the results of a cross-cultural adaptation of previously validated measures of children’s movement behaviors and parenting practices in economically disadvantaged Brazilian families. Study 1 involved translation of the instruments from English to Portuguese. A team of translators (fluent in both English and Portuguese) and researchers followed established procedures for translating measurement scales, identifying problematic items, and reaching consensus on discrepancies. Study 2 involved cognitive interviews with 24 parents from urban and rural North-eastern Brazil addressing the format, content, and clarity of the items. Half the parents provided feedback on the first 33 items of the questionnaire, with the remaining parents providing feedback on the final 29 items. Notes were recorded during the interview and parents’ feedback summarized in a report. In the translation and back-translation, 15 discrepancies were identified. These were mostly due to multiple Portuguese words having the same meaning in English. The research team discussed these discrepancies and consensus was reached to ensure that the concepts depicted in the Portuguese version were consistent with the English version. In the cognitive interviews, parents identified minor problems with item comprehension resulting in minor adaptations to response options, recall period, and format of the questionnaire. The process of translation and cognitive interviews conducted in Brazilian families resulted in an appropriate cultural adaptation of scales measuring children’s movement behaviors and parenting practices. Future studies should evaluate the validity and reliability of the measures in LMIC families.

## 1. Introduction

Childhood obesity has become one of the most serious health challenges worldwide. Globally, it is estimated that over 41 million children aged 0 to 5 years are overweight or obese [1]. Low-to-middle income countries (LMIC) such as Brazil are disproportionately affected by childhood obesity. In 2016, 44% of children under 5 from LMIC were overweight or obese, an increase from 10 million to 15.6 million from 2000 to 2016 [2]. In north-east Brazil, the prevalence of being overweight and obese among children under 5 years of age is estimated to be 34% or 1 in 3 children [3]. This is of concern due to the significant impacts on health and well-being. Young children who are overweight or obese are more likely to experience significant short-term health problems, including asthma, sleep apnea, elevated blood pressure, musculoskeletal disorders, fatty liver disease, and insulin resistance [4,5,6]. Compared to peers with a healthy weight, overweight and obese children often experience bullying and teasing at school and have significant mental health issues, such as depression, anxiety, and disordered eating [4,5,6]. The long-term health impacts of obesity in childhood include, among others, heart disease and stroke, type 2 diabetes, and the development of some cancers (i.e., endometrial and breast) [7]. Moreover, obesity in childhood increases the risk of obesity in adulthood [8].

Regular physical activity, limited screen time, and adequate sleep are key movement behaviors that contribute to the prevention of obesity in young children [9,10,11]. The World Health Organization recommends that children aged 3 to 5 spend a minimum of 3 h a day in active play, with 60 min of moderate to vigorous physical activity (MVPA); accumulate less than 60 min of sedentary recreational screen time; and sleep between 10 to 13 h daily [12]. Currently, the proportion of South American children under 5 meeting this recommendation is not clear. When physical activity is objectively measured using an accelerometer, between 12.7% and 100% of South American children meet the daily 60 min MVPA, with estimates varying according to method used to categorize MVPA [13]. Based on parent reported data, the percentage of young children exposed to at least 2 h per day of screen time ranges from 39.4% to 100% [13]. Currently, there are no published data regarding the proportion of South American children under 5 meeting sleep recommendations.

Parents are key socialization agents in the establishment of healthful movement behaviors in young children [9,14,15]. Parenting practices are the strategies parents use to assist or support children in their socialization goals, including the establishment of healthy movement behaviors [16]. A number of parenting practices, including logistic support, co-participation, modelling, and setting rules and limits play a crucial role in the development of healthy movement behaviors in young children [17,18,19]. However, to date, the evidence related to effective parenting is primarily from studies conducted in families from high-income countries and little is known about the relationship between parenting practices and children’s movement behaviors in LMIC communities. Investigations into the relationship between parenting practices and children’s movement behaviors need to consider the cultural context in which families reside, and how the physical and social environments, particularly poverty, influence the relationships between the parent and the child [20]. However, no studies have been conducted in LMIC families because of the lack of validated culturally appropriate measurement scales.

The process of culturally adapting measurement scales validated in other population groups encompasses translation into the language of interest and the application of cognitive interviewing [21]. A rigorous protocol for translating a measurement scale from one language to another is vital in ensuring that the translated version is as close as possible to the original version of the scale [22]. Cognitive interviewing obtains feedback from respondents about the items’ content, format, and comprehension of response options to improve participant recall [23]. To date, no previous study has adapted instruments measuring children’s movement behavior and associated parenting practices in families living in Brazil, or any other LMIC. Identifying and understanding the parenting practices that support healthful movement behaviors in young children from LMIC families is necessary for the development of effective interventions to prevent childhood obesity. This paper reports the cross-cultural adaptation of previously validated measures of children’s movement behaviors and parenting practices. A brief description of each measure appears in Table 1. Study 1 addressed the translation of the measures from English to Portuguese, while Study 2 used cognitive interviewing to determine if the format, content, and clarity of the items were adequate for use among Brazilian families. 

## 2. Materials and Methods—Study 1

The objective of Study 1 was to translate measurement tools assessing children’s movement behaviors and parenting practices that were originally developed and validated in English into Portuguese.

Following the procedures outlined by Beaton and colleagues, [22] the English version of the scales was first translated into Portuguese by the principal investigator and another Portuguese speaking researcher who was not aware of the concepts being studied. After the initial translation, the two translators and a third independent researcher, fluent in Portuguese and English, compared the translated versions of the scales to identify items with discrepancies, discuss the discrepancies with the two translators, and reach consensus on the wording of items. The preliminary Portuguese version of the questionnaire was then translated back into English by two independent Brazilian researchers (fluent in both English and Portuguese). This version was compared with the original instrument by an “expert committee”, comprising the principal investigator (WSFG) and members of the research team (RB and SGT). Discrepancies were identified, discussed, and consensus reached on the final wording.

## 3. Results—Study 1

After the initial translation, six discrepancies were identified, discussed, and resolved. The discrepancies are reported in Table 2. Discrepancies (highlighted in bold type) were mostly due to multiple words in the Portuguese language having the same meaning in English, or the absence of an equivalent word in Portuguese, such as the expressions “*calm down*” and “*get tucked in*”. These discrepancies in translations were discussed, and consensus was reached with respect to the final wording.

The expert committee identified seven discrepancies (highlighted in bold type) between the translated-back version and original English version of the questionnaire. The discrepancies are summarized in Table 3. Discrepancies were again mostly due to multiple Portuguese words having the same meaning in English. A number of missing words in the re-translation were also identified and corrected, including the words “separated”, “phones and tablets”, “how much” and “in the hour”. Additionally, even though the abbreviations “a.m.” and “p.m.” are used informally in Brazil, they could not be directly translated into Portuguese and were changed to “morning” and ”night”. After confirmation that the instrument translated into Portuguese presented the same concepts of the English version, the Portuguese version questionnaire was finalized in preparation for the cognitive interview study.

## 4. Materials and Methods—Study 2

The objective of Study 2 was to determine if the format, content, and clarity of the items were adequate for use among Brazilian families. A total of 68 parents or carers (hereafter referred to as parents) from two early childhood education and care (ECEC) centers from rural and urban communities from Caruaru, Brazil were invited to participate in the study. This region is home to approximately 361,000 people with 36% of them having a monthly income half of the minimum Brazilian wage (equivalent to 85 USD), and only 7.7% having completed higher education [29,30].

Prior to participation in the study, parents provided written or, in the case of low literacy levels, verbal consent. The research was approved by the Human Research Ethics Committee of the Queensland University of Technology, Brisbane/Australia (Approval No. 1800001141) and the Department of Education of Caruaru, Brazil (Approval Letter dated 1 March 2019). The study was completed between March and April 2019.

Parents completed the questionnaire individually as an interviewer-administered survey. All interviews were conducted on-site at the ECEC by the principal investigator. Fifty percent of the parents provided feedback on the first half of the questionnaire (33 items), with the remaining parents providing feedback on items from the second half of the questionnaire (29 items). The parents who were interviewed from Monday to Wednesday morning completed the first half of the survey. The parents who were interviewed from Wednesday afternoon to Friday completed the second half of the survey. The list of prompts used during the cognitive interviews (see Supplementary file 1), included discussion about the format of the questionnaire, addressing font size, type, and readability [23]. The content discussion consisted of checking the parents’ understanding of the item by asking them to “think out loud” while providing a response, the appropriateness of the responses and response format, and general comments about the complexity of the items [23]. Notes were recorded during the interview and the parent’s feedback was summarized in a report. The research team then reviewed this report to identify items that required modifications.

## 5. Results—Study 2

Of the 68 parents at the two centers, 33 agreed to participate. However, 9 parents did not attend the interview, resulting in the participation of 24 parents (12 parents from each ECEC). The World Health Organization recommends the participation of at least 10 participants per section of an instrument for the process of translation and adaptation of instruments [21]. Each interview took approximately 45 min to complete. The majority of parents were female (83%), aged between 25 to 35 years, and were married or living with a partner (66%). Seventy-five percent of the parents were earning up the minimum Brazilian wage (up to 170 USD), and only 25% of them had completed elementary school.

Parents reported that they understood most of the questions and response options. The complete list of issues identified, and modifications are described in Table 4. Parents indicated that reporting the duration of children’s screen time was difficult. First, they struggled to understand the term “usual”, as the word “usual” is not regularly used in low-educated Brazilian families. The solution was to change this term to “*normal*” for ease of understanding. In addition, the screen time items required parents to estimate the total amount of screen time across five weekdays and two weekend days for different types of screen time. However, parents had difficulty recalling and adding up daily totals (number of hours and minutes) to come up with a total amount across all weekdays and weekend days. This problem was addressed by asking parents to estimate the *daily* amount of screen time on a normal weekday and weekend day for each digital device. Parents also reported that reporting screen time hours for television separately from time spent watching DVDs and videos was confusing as children watched videos and DVDs on the television. To alleviate the confusion, time spent watching television and watching DVDs and videos was combined into a single item. To make it more convenient for parents to report screen time, the responses to items asking about time spent watching or using digital devices were placed side by side in a table, avoiding the need for separate items for weekdays and weekend days.

In addition to these wording issues, those parents who were able to read and complete the questionnaire suggested several formatting changes to improve understanding of items, including additional white space between sentences to improve readability and putting key words in italics, underline or in bold type to emphasize importance. Parents also indicated that the wording of the instructions for some of sections could be reduced or combined with other sections. Parents also proposed displaying the response choices horizontally across the page rather than vertically like a multiple-choice exam question. A consistent recall period for all survey items was also suggested, such as a normal day over the last month. An additional two response options were suggested and added regarding parents’ educational level and employment status, including” post-graduation” and “retired”, respectively. For some Likert type items, the response categories capturing the frequency or likelihood of an event or behavior (i.e., occasionally, anytime, constantly) were difficult for parents to comprehend, and were replaced by “sometimes” and “always”. Moreover, the word “events” was viewed as ambiguous and replaced by “activities” or “actions”. Finally, parents did not understand the term sedentary. Therefore, whenever the word, “sedentary” was used in the questionnaire, examples of this behavior were given including “not active” and “sitting for a long time”. The final version of the Portuguese and English measurement scales, and details of scoring are available as supplementary content (see Supplementary files 2–4, respectively).

There were some changes suggested by the participants that could not be implemented. In relation to Likert-type items with responses ranging from “strongly disagree” to “strongly agree”, parents suggested simplifying response options to only “agree” or “disagree”. Parents also indicated that having the same response options across the entire survey would improve their understanding. However, these changes were not implemented by the research team because they would fundamentally change the characteristics of the original measurement scales e.g., a Likert scale measuring frequency is not interchangeable with response categories assessing agreement.

## 6. Discussion

This paper described the results of two studies undertaken to translate and adapt previously validated measures of children’s movement behaviors and parenting practices for use among Brazilian families. To our knowledge, it is the first study to culturally adapt scales measuring children’s movement behaviors and parenting practices in Brazil, or any other LMIC. Rigorous protocols to translate the instrument from English to Portuguese and create measures that were culturally appropriate were implemented. Minor discrepancies between translators were identified, discussed, and consensus was reached, resulting in a final Portuguese version of the questionnaire. In the cognitive interviews, parents understood most items, but requested modifications to the formatting of the questionnaire, recall period, and the wording of a small number of items.

In the initial translation process from English to Portuguese, several discrepancies between translators were identified. Most of them could be explained by the considerable number of words in Portuguese that have the same meaning in English, or the absence of an equivalent word in Portuguese. These results highlight the value of going through a rigorous process of translating the measurement scales, as these discrepancies were not anticipated and would have not been detected if only one person translated the measures from English to Portuguese. Moreover, even after agreement was reached between translators, scrutiny from expert researchers identified some important differences between the back translation into English and the original measures. Conducting these processes led to an accurate translation of the measurement scales to be applied in Brazilian families.

Parents provided valuable feedback about the wordiness and format of the survey. For ease of readability, parents suggested modifications to the format of the questionnaire, including the addition of space between sentences and emphasizing important words in each item. Problematic items, including those requiring responses related to frequency/likelihood, such as “occasionally” and “anytime” were adapted based on the parent’s suggestions for alternatives words, using their own vocabulary to improve clarity. Standardizing the recall period for all scales to “a normal day, considering the last month”, was also a key suggestion made by parents. These adaptations highlight the need to conduct cognitive interview studies before new or adapted measurement scales are deployed in other cultural settings.

A key finding of the current study was the difficulty associated with estimating and aggregating screen time over all five weekdays and two weekend days. First, parents did not understand the term “*usual*”, and this was changed to “*normal*". Second, most parents lacked the numeracy skills required to recall and sum their child’s exposure to screen time over multiple days. To improve recall and enhance accuracy, parents suggested reporting *daily* exposure to screen time on a *normal* weekday and on a *normal* weekend day, rather than reporting aggregate exposure to screen time over multiple days. Parents also reported difficulties differentiating screen time watching television from watching DVDs or videos. To minimize the burden, the total number of hours spent watching television, DVDs or videos were combined into a single item. This modification simplified the cognitive task of summing estimates of screen time from different modalities. This finding underscores the importance of rigorously testing and adapting measurement scales prior to deploying them in study populations with low numeracy and literacy levels.

The current study has several strengths. First, parents were recruited from urban and rural areas with low levels of numeracy and literacy representative of families in the region. In addition, established protocols for culturally adapting measurement scales in relation to translation from English to Portuguese and cognitive interviewing were followed [21,22,23]. The procedures undertaken to interview these parents were resource and time intensive, requiring a highly skilled interviewer to listen to parent’s feedback and document their suggestions accurately. Offsetting these strengths, there were several limitations. First, this study was conducted in one rural and one urban province in Brazil. Therefore, the results may not be generalizable to all of Brazil and other LMIC communities. Moreover, there were some changes suggested by participants that could not be implemented, after the expert committee concluded that such changes would significantly alter the intent of the original questions. However, such occurrences were rare and most of the feedback provided by parents was actioned. Finally, most of the participants completing the interviews were mothers. However, this is not uncommon in parenting studies [31,32].

## 7. Conclusions

In conclusion, the translation and cognitive interviewing processes undertaken in Brazilian families resulted in an appropriate cultural adaptation of measurement scales assessing children’s movement behaviors and associated parenting practices. The findings highlight the importance of undertaking appropriate steps to ensure that prior to use in any investigation, measures developed for other cultures are carefully translated and tested for understanding in the target study population. Future studies should evaluate the psychometrics properties of the adapted scales in families living in LMIC countries, including Brazil. With supporting evidence of reliability and validity, the measures could then be used to examine the relationships between parenting practices and children’s movement behaviors in LMIC communities, informing the development of family-based interventions to improve movement behaviors, and prevent obesity in young children.

## Figures and Tables

**Table 1:** Measurement scales selected for the cross-cultural translation process.

Construct	Instrument	Description	Sample Items
*Physical Activity*	Outdoor Playtime Recall Burdette et al. [24]; validity (r = 0.33) against accelerometry	Duration of play: weekday and weekend days	How much time would you say your child spends playing outdoors on a typical weekday/weekend day?
*Screen Time*	InFANT Hesketh et al. [25]	Duration of screen time: weekday and weekend days	During a usual week, how much time does your child spend doing each of the following at home? Watching television programs, DVDs or videos; using a computer, etc.
*Sleep*	POI.nz Taylor et al. [26]	Total sleep duration: weekday and weekend days	What time does your child usually fall asleep at night/wake up in the morning to start the day?
*Physical Activity*	Physical Activity Parenting Practices Vaughn et al. [27]; internal consistency = 0.54–0.88	Controlling and supportive physical activity parenting practices	How often do you use sports or physical activities to get your child to do something? How often do you use your own behavior to encourage your child to be physically active?
*Screen Time*	Physical Activity Parenting Practices Vaughn et al. [27]; internal consistency = 0.66–0.79	Controlling and Supportive screen parenting practices	About how much time is your child allowed to watch each weekday/weekend day? How much do you enjoy watching TV or movies during your free time?
*Sleep*	Bedtime Routines Questionnaire Henderson et al. [28]; factors loadings = 0.50–0.94	Child bedtime and pre-bed activities	During weeknights/weekend nights for the past month, how often did your child perform the same activities? In the past month, in the hour before going to bed, how often did your child read/listen to a story?

**Table 2:** Discrepancies in the translations of the measurement tools in English to Portuguese.

Original Version	Translator 1 Version	Translator 2 Version	Final Version
How often do you ask your child to **calm down** their indoor play?	How often do you ask your child to **stay quiet** their indoor play?	How often do you ask your child to **quiet down** their indoor play?	How often do you ask your child to **quiet down** their indoor play?
How often do you **take away TV**, video, or movie time as a punishment for bad behavior?	How often do you **take off TV** time, video, or movie time as a punishment for bad behavior?	How often do you **do not let** your child watch TV, video, or movie time as a punishment for bad behavior?	How often do you **take off TV** time, video, or movie time as a punishment for bad behavior?
How often do you **take outside time away** from your child for bad behavior?	How often do you **take off outside time** from your child for bad behavior?	How often do you **reduce the time** from your child for bad behavior?	How often do you **take off outside time** from your child for bad behavior?
**How valuable** is it to you that your child be physically active?	**How important** is it to you that your child be physically active?	**How valuable** is it to you that your child be physically active?	**How valuable** is it to you that your child be physically active?
During a typical week, how often do you **send your child** outside to play so you can get things done around the house?	During a typical week, how often do you **ask your child** outside to play so you can get things done around the house?	During a typical week, how often do you **send your child** outside to play so you can get things done around the house?	During a typical week, how often do you **send your child** outside to play so you can get things done around the house?
In the past month, in the hour before going to bed, how often did your child **get tucked in**?	In the past month, in the hour before going to bed, how often did your child **was put to bed**?	In the past month, in the hour before going to bed, how often did your child **was placed in bed**?	In the past month, in the hour before going to bed, how often did your child **was put to bed**?

**Table 3:** Discrepancies in the back-translated version of the measurement tools.

Original Version	Translator 1 Version	Translator 2 Version	Final Version
When inside the house, my child can use toys and equipment for **physically active play**	When inside the house, my child can use toys and equipment for **physical activities**	When inside the house, my child can use toys and equipment for **physically active games**	When inside the house, my child can use toys and equipment for **physically active games**
How often do you ask your child to **calm down** their indoor play?	How often do you ask your child **to be quiet** their indoor play?	How often do you ask your child **to quiet down** their indoor play?	How often do you ask your child **to quiet down** their indoor play?
How often do you go to your child’s sporting events, lessons, or other organized physical activities with them (e.g., watch your child perform in a dance recital, swim meets, or **practice**)?	How often do you go to your child’s sporting events, lessons, or other organized physical activities with them (e.g., watch your child perform in a dance recital, swim meets, or **training sessions**)?	How often do you go to your child’s sporting events, lessons, or other organized physical activities with them (e.g., watch your child perform in a dance recital, swim meets, or **training**)?	How often do you go to your child’s sporting events, lessons, or other organized physical activities with them (e.g., watch your child perform in a dance recital, swim meets, or training sessions)?
I can **get** my child to be physically active at home	I can **make** my child to be physically active at home	I can **get** my child to be physically active at home	I can **get** my child to be physically active at home
**Others adults in my child’s life** make it hard to enforce household rules about TV viewing	**Other adults who have contact with my child** make it hard to enforce household rules about TV viewing	**The others adults who live with my child** make it hard to enforce household rules about TV viewing	**Other adults who have contact with my child** make it hard to enforce household rules about TV viewing
During a typical week, how often do you **send** your child outside to play so you can get things done around the house?	During a typical week, how often do you **ask** your child outside to play so you can get things done around the house?	During a typical week, how often do you **send** your child outside to play so you can get things done around the house?	During a typical week, how often do you **send** your child outside to play so you can get things done around the house?
During a typical week, how often does your child hear you say that you were too tired **to be active**?	During a typical week, how often does your child hear you say that you were too tired **to exercise**?	During a typical week, how often does your child hear you say that you were too tired **to do physical activities**?	During a typical week, how often does your child hear you say that you were too tired **to do physical activities**?

**Table 4:** Problems and solutions that were identified in cognitive interviews.

First Version	Problem	Revised Version
***Demographic Questions***		
This section is about your child **personal** information	Difficulty comprehending the term “personal”	This section of the survey is **about your child**
**Less or equal than** 24 years old	Unusual sentence in the Brazilian context	**Up to** 24 years old
Employed **full-time**	Difficulty comprehending the term “full-time”	Employed full-time-**full day**
What was the income range of the **child’s family** in the last month?	The word “child’s” were reported to be unnecessary	What was the income range of the **family** in the last month?
How many people live **with you? (including the child)**	Unsure whether to count self in the total	How many people live in **your house? (including you)**
Response options regarding **parents’ educational level and employment status** were added.	An additional two possible responses were suggested by parents.	Options of **post-graduation and retired** were added.
***Parenting practices questions***		
About your **interactions with your child**	Difficulty comprehending what the sub-heading were about	About your interactions with your child **in relation to physical activity and screen time**
You can’t go outside to play until you eat your **peas**	Brazilian parents use another type of food, or total amount eaten as a bribe	You can’t go outside to play until you eat your **lunch**
**Please read each of the following statements and then place an “X”** in the box that best describes how much you agree or disagree with that statement	The term “Please read each of the following statements” were reported to be unnecessary	**Place an “X”** in the box below that best represents how much you agree or disagree with each of the following statements
**For the following items,** place an “X” in the box that best represents how often each of the following things happen during a **typical** week	The term “For the following items” were reported to be unnecessary; Difficulty understanding what a typical week was	**Place an “X”** in the box below that best represents how often each of the following things happen during a **normal** week
About how much time is **s(he)** child allowed to watch/play videogames/each weekday/weekend day?	Difficulty comprehending the term “s(he)”	About how much time is **your** child allowed to watch/play videogames/each weekday/weekend day?
If you don’t stop that you will not be able to go to **Karate**	Karate is not a common sport in the Brazilian context	If you don’t stop that you will not be able to go to **soccer/dance class this afternoon**
**Ice skating** as an example	Ice skating cannot be performed in Brazil	**Playing soccer/volleyball**
**How much** do you use your own behavior to encourage your child to be active?	Difficulty comprehending the term “how much”	**How often** do you use your own behavior to encourage your child to be physically active?
**How active** are you in enrolling your child in sports?	Difficulty comprehending the term “how active”	**How often** do you enroll your child in sports?
**I go out of my way** to enroll my child in sports	Difficulty comprehending the term “I go out of my way”	**I always** enroll my child in sports
During the last month, how many times have you taken your child to play at a **park**?	Park is not an existing common place in rural areas of Brazil	During the last month, how many times have you taken your child to play at a park, **playground, or other leisure area of the region**?
**Others adults in my child’s life** make it hard to enforce household rules **about TV viewing**	Difficulty comprehending the sentence	**Others adults who have contact with my child** make it hard to enforce household rules **about how much time she/he is allowed to watch TV**
Do you tell your child how **sedentary habits can be unhealthy**?	Difficulty comprehending the term “sedentary” and “unhealthy”	Do you tell your child how sedentary habits **(for example, sitting for a long time) can be harmful to health**?
Do you send your child outside to play so you can **get things done around the house**?	Difficulty comprehending the term “get things done around the house”	Do you send your child outside to play so you can **do household chores in your home**?
Does your behavior encourage your child to be **sedentary**?	Difficulty comprehending the term “sedentary”	Does your behavior encourage your child to be sedentary (**for example, watching TV for a long time**)?
Does your child hear you say that you were too tired **to be active**?	Difficulty comprehending the term “to be active”	Does your child hear you say that you were too tired **to do physical activities**?
Do you say things to encourage your child to spend less time being **sedentary**?	Difficulty comprehending the term “sedentary”	Do you say things to encourage your child to spend less time being sedentary? (**for example, stop watching and go play**)
How important is it **for your child**…?	Difficulty comprehending the context of the question	How important is it **for you that your child**…?
During weeknights for the past month, how often did your child… go to bed at the same time (within 10 min)?	Parents were unsure of what the term “within 10 minutes” referred about	During weeknights for the past month, how often did your child… go to bed at the same time (within 10 min **of the scheduled time**)?
During weeknights (**Sunday through Thursday nights only**) for the past month	Difficulty comprehending the meaning of “Sunday through Thursday nights only”	**During weeknights for the past month**
During weeknights (**Friday and Saturday nights**) for the past month	Difficulty comprehending the meaning of “Friday and Saturday nights”	**During weekend nights for the past month**
**Hug/kiss caregiver**?	Difficulty comprehending the differences between “Hug/kiss caregiver” and “Cuddle with caregiver”	Hug/kiss caregiver (**for example, a goodnight kiss**)?
**Cuddle with caregiver**?	Difficulty comprehending the term “cuddle”	Cuddle with caregiver (**for example, the child sat on the lap of the caregiver and was hugged by him/her**)?
Response categories with “**constantly**” and “**anytime”**	Difficulty comprehending “constantly” and “anytime”	Replaced by “**always**”
Response category with “**occasionally**”	Difficulty comprehending the term “occasionally’	Replaced by “**sometimes**”
The word “**events**”	The word “events” was viewed as ambiguous	Replaced by “**activities**” or “**actions”**
***Movement behaviors questions***		
A **typical or usual** weekday/weekend day	Difficulty understanding typical or a usual weekday and weekend day	A **normal** weekday/weekend day/week
Time spent on watching TV and DVDs or videos were reported **separately**	Difficulty reporting time spent on watching TV and watching DVDs or videos	Time spent on watching TV and DVDs or videos were **combined**
The following questions **ask about your** child’s sleep routine **over the last 2 weeks**	Difficulties having different recall periods across the survey	The following questions **are related to** your child’s sleep routines **over the last month**

## Data Availability

Data sharing is not applicable to this article as no datasets were generated or analyzed during the current study. However, the Portuguese and English measurement scales and details of scoring are available in this article as a supplementary content.

## Supplementary Materials

The following are available online at https://www.mdpi.com/1660-4601/18/1/239/s1, Supplementary File 1: list of prompts used during the cognitive interviews, Supplementary File 2: Portuguese version of the measurement scales, Supplementary File 3: English version of the measurement scales, Supplementary File 4: Calculation for the measurement scales.
