# Supplementary material for: Cross-Cultural Adaptation of Instruments Measuring Children’s Movement Behaviors and Parenting Practices in Brazilian Families
Source: Int J Environ Res Public Health. 2020 Dec 31;18(1):239. doi: 10.3390/ijerph18010239 (PMC7794996; doi:10.3390/ijerph18010239)
Supplement: Supplementary file 1 [file ijerph-18-00239-s001.zip › Supplementary File 1.docx]

Table 1. List of prompts used during the cognitive interviews.

| **General probes** |
| --- |
| What did you think of the questionnaire? |
| Was the size of the words easy to read? |
| Was the format of the questionnaire easy to follow to? |
|  |
| **Questions per section:**  **Demographics; Child Behavior; Parent Practices** |
| What did you think about this section? |
| Was it easy or difficult to answer the questions? |
| What did you think of the instructions? Was it useful? |
| Are there any questions or words that were not clear? |
| How was it for you to go through that list? Did that cause any difficulties? |
| What do you think about the response choices? |
| What does "in the past 30 days" mean to you? |
| How did you calculate your answer for this particular question? |
|  |
| **Potential Prompts** |
| Could you explain why this question was not clear? |
| How we could make it clearer? |
| How would you make the response choices clearer or easier to understand? |
| Why do you say/think that? |
