# Supplementary material for: Cross-Cultural Adaptation of Instruments Measuring Children’s Movement Behaviors and Parenting Practices in Brazilian Families
Source: Int J Environ Res Public Health. 2020 Dec 31;18(1):239. doi: 10.3390/ijerph18010239 (PMC7794996; doi:10.3390/ijerph18010239)
Supplement: Supplementary file 1 [file ijerph-18-00239-s001.zip › Supplementary File 2_Without Scoring.docx]

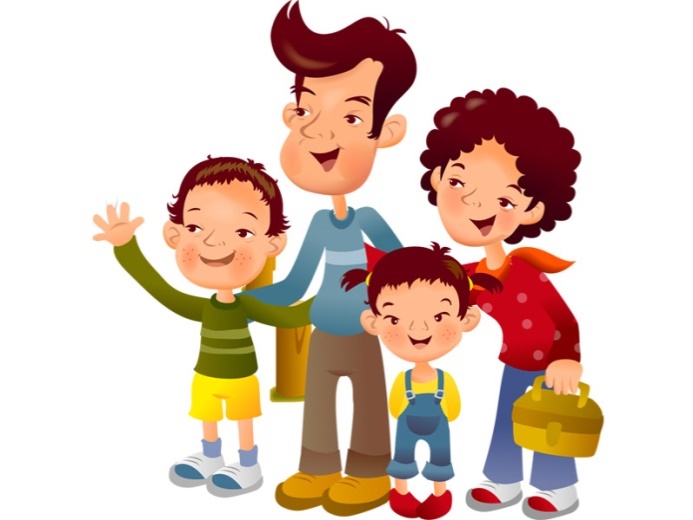


Questionário Saúde da Família

| - O objetivo deste questionário é obter informações sobre o seu comportamento de saúde e o comportamento de saúde da sua criança.      - Por favor, responda as questões apenas sobre a sua criança mais velha entre 3 e 6 anos de idade que esteja matriculado na creche/pré-escola. - Seja o mais preciso possível – não há respostas certas ou erradas. - Todas as informações são rigososamente confidenciais. - Por favor, tente responder a todas as perguntas e forneça apenas uma resposta para cada questão. - Caso você não tenha certeza de como responder alguma questão, peça ajuda à equipe de pesquisa. - Suas respostas são muito importantes para nós. - Responder à este questionário tomará cerca de 45 minutos do seu tempo. |
| --- |

Identificação

Nome da Criança: ______________________________________________________________________

Creche/Pré-escola: _____________________________________________________________________

Série: ( ) Berçario II ( ) Pré I ( ) Pré II

Participante ID: __________

Creche ID: __________

| ***Informações Pessoais da sua Criança*** |
| --- |

*Esta parte do questionário é sobre a sua criança.*

1. Qual é o sexo da sua criança?

🞎 Feminino

🞎 Masculino

1. Qual é a data de nascimento da sua criança?

**_______ / _______ / _______**

dia mês ano

1. Qual é a raça/cor da sua criança?

🞎 Branco(a)

🞎 Amarelo(a)

🞎 Pardo(a)/mestiço(a)

🞎 Preto(a): moreno(a)/negro(a)

🞎 Indígena

1. Qual o período que a sua criança frequenta a creche/pré-escola?

🞎 Meio período (manhã ou tarde)

🞎 Período integral (manhã e tarde)

| ***Sobre você...*** |
| --- |

*Esta parte do questionário é sobre você e sua família.*

1. Quantos anos você tem?

🞎 Até 24 anos

🞎 Entre 25 e 35 anos

🞎 Entre 36 e 45 anos

🞎 Mais do que 46 anos

1. Qual a sua relação com a criança?

🞎 Adulto responsável do sexo feminino (ex.: mãe, madrasta, avó, tia)

🞎 Adulto responsável do sexo masculino (ex.: pai, padrasto, avô, tio)

1. Qual é o seu nível mais alto de escolaridade?

🞎 Não tenho estudos

🞎 Ensino fundamental incompleto

🞎 Ensino fundamental completo

🞎 Ensino médio incompleto

🞎 Ensino médio completo

🞎 Ensino superior incompleto

🞎 Ensino superior completo

🞎 Pós-graduação

1. Qual é o seu estado civil?

🞎 Solteiro(a)

🞎 Casado(a)

🞎 Morando com o companheiro(a)

🞎 Separado(a) ou divorciado(a)

🞎 Viúvo(a)

1. Como você descreve sua condição de emprego hoje?

🞎 Empregado em período integral ‘o dia todo’ (incluir trabalhando como autônomo)

🞎 Empregado em meio período (incluir trabalhando como autônomo)

🞎 Trabalho casualmente

🞎 Não trabalho devido a problemas de saúde

🞎 Desempregado

🞎 Aposentado

1. Qual foi a faixa de renda da família no último mês?

🞎 Até R$998

🞎 Entre R$998 e R$1497

🞎 Entre R$1498 e R$1996

🞎 Maior do que R$1997

1. Você recebe apoio financeiro do governo por meio do ‘Programa Bolsa Família’ por sua criança frequenter a creche/pré-escola?

🞎 Sim

🞎 Não

1. Quantas pessoas moram na sua casa? (incluindo você).

🞎 Um

🞎 Dois

🞎 Três

🞎 Quatro

🞎 Cinco ou mais

As perguntas a seguir são sobre a atividade física semanal da sua criança.

Pense um momento sobre um dia de semana normal da sua criança no último mês.

1. Quanto tempo você diria que a sua criança passa brincando ao ar livre em um dia normal da **semana**?

Horas: Minutos:

Agora pense um momento sobre um dia de final de semana normal da sua criança no último mês*.*

1. Quanto tempo você diria que a sua criança passa brincando ao ar livre em um dia normal de **final de semana**?

Horas: Minutos:

| ***Sobre o Tempo de Tela da sua Criança*** |
| --- |

*As perguntas a seguir são sobre o tempo de tela semanal da sua criança. Isto inclui o uso de televisão, iPads/tablets, telefones celulares, videogames e computadores.*

| ***Sobre a Atividade Física da sua Criança*** |
| --- |

1. Pensando no último mês, quanto tempo a sua criança gasta fazendo cada uma das seguintes atividades em casa? Por favor, informe separadamente para os dias da semana e para os dias do final de semana.

|  | Em um dia normal da **semana**  Segunda - Sexta  horas/minutos | Em um dia normal do **final de semana**  Sábado e Domingo  horas/minutos |
| --- | --- | --- |
| **A. Assistindo TV/DVDs** |  | **mins**  **hrs** |
| **B. Usando o computador** | **mins**  **hrs**  **mins**  **hrs** | **hrs**  **mins** |
| **C. Jogando videogame (e.g. Nintendo DS, Playstation, Xbox, etc.)** | **mins**  **hrs** | **hrs**  **mins** |
| **D. Usando telefone celular e Ipads/Tablet** | **mins**  **hrs** | **hrs**  **mins** |

*As perguntas a seguir estão relacionadas as rotinas de sono da sua criança.*

| ***Sobre o Sono da sua Criança*** |
| --- |

Pensando no último mês...

1. Que horas a sua criança geralmente dorme a noite?

Em um dia normal da **semana**: Horas: Minutos:

Em um dia normal do **final de semana**: Horas: Minutos:

1. Que horas a sua criança geralmente acorda de manhã para começar o dia?

Em um dia normal da **semana**: Horas: Minutos:

Em um dia normal do **final de semana**: Horas: Minutos:

*As perguntas a seguir são sobre como você interage com sua criança em relação a atividade física e tempo de tela.* Estamos interessados no que **você** faz e como **você** se sente. Por favor, leia cada questão e selecione a melhor resposta para você. Leve o tempo que precisar e responda com a maior precisão possível.

| ***Sobre suas Interações com a sua Criança em Relação a Atividade Física e Tempo de Tela*** |
| --- |

1. Com que **frequência** a TV da sua casa fica ligada quando tem pessoas em casa?

🞎 Muito raramente

🞎 Raramente

🞎 Às vezes

🞎 Frequentemente

🞎 Muito frequentemente

🞎 Sempre

1. Marque um “X” no quadrado abaixo que melhor representa a frequência com que a sua criança faz cada uma das seguintes atividades enquanto brinca **dentro de sua casa.**

|  | **Sempre** | **Algumas vezes** | **Nunca** |
| --- | --- | --- | --- |
| 1. Saltitar, pular, galopar | 🞎 | 🞎 | 🞎 |
| 1. Correr | 🞎 | 🞎 | 🞎 |
| 1. Brincar de pega-pega | 🞎 | 🞎 | 🞎 |
| 1. Brincar se rolando no chão (lutar no chão) | 🞎 | 🞎 | 🞎 |
| 1. Saltar de um lugar mais alto | 🞎 | 🞎 | 🞎 |
| 1. Dar cambalhota (pulando ou caindo) | 🞎 | 🞎 | 🞎 |
| 1. Escalar | 🞎 | 🞎 | 🞎 |
| 1. Se balançar ou ficar pendurado | 🞎 | 🞎 | 🞎 |
| 1. Se equilibrar | 🞎 | 🞎 | 🞎 |
| 1. Juntar travesseiros e pular sobre eles | 🞎 | 🞎 | 🞎 |
| 1. Lançar, jogar ou chutar uma bola | 🞎 | 🞎 | 🞎 |

1. Marque um “X” no quadrado abaixo que melhor representa o quanto você concorda ou não concorda com cada uma das seguintes frases.

|  | **Não concordo totalmente** | **Não concordo** | **Não concordo e nem discordo** | | **Concordo** | **Concordo totalmente** |
| --- | --- | --- | --- | --- | --- | --- |
| 1. Quando minha criança está **dentro de casa**, ele/ela deve brincar de forma quieta sem fazer muito barulho. | 🞎 | 🞎 | 🞎 | 🞎 | | 🞎 |
| 1. Quando **dentro de casa**, minha criança pode usar brinquedos e equipamentos para jogos fisicamente ativos (*por exemplo, atividades motoras como correr, saltar, pular ou dar cambalhotas*). | 🞎 | 🞎 | 🞎 | 🞎 | | 🞎 |

1. Com que **frequência** você pede a sua criança para se aquietar quando ele/ela está brincando **dentro de casa**?

🞎 Nunca

🞎 Muito raramente

🞎 Raramente

🞎 Algumas vezes

🞎 Frequente

🞎 Muito frequente

1. Marque um “X” no quadrado abaixo que melhor representa a frequência com que você faz cada uma das seguintes coisas relacionadas ao **jogo ao ar livre** da sua criança.

| **Com que frequência você…** | **Nunca** | **Muito raramente** | **Raramente** | **Às vezes** | **Frequente** | **Muito frequente** |
| --- | --- | --- | --- | --- | --- | --- |
| a. pede a sua criança para **não** correr quando ele/ela brinca **fora de casa**? | 🞎 | 🞎 | 🞎 | 🞎 | 🞎 | 🞎 |
| b. pede a sua criança para tentar manter-se limpa quando ele/ela brinca **fora de casa**? | 🞎 | 🞎 | 🞎 | 🞎 | 🞎 | 🞎 |
| c. deixa a sua criança brincar **fora de** **casa** em dias quentes? | 🞎 | 🞎 | 🞎 | 🞎 | 🞎 | 🞎 |
| d. deixa a sua criança brincar **fora de** **casa** em dias frios? | 🞎 | 🞎 | 🞎 | 🞎 | 🞎 | 🞎 |
| e. pede a sua criança para se aquietar quando ele/ela brinca **fora de casa**? | 🞎 | 🞎 | 🞎 | 🞎 | 🞎 | 🞎 |
| f. pede a sua criança para não sujar a roupa quando ele/ela brinca **fora de casa**? | 🞎 | 🞎 | 🞎 | 🞎 | 🞎 | 🞎 |

1. Você limita a quantidade de tempo que a sua criança assiste TV, vídeos ou filmes **durante a semana** (segunda a sexta-feira)?

🞎 Sim

🞎 Não →

**Se "NÃO", pule para a questão 25**

1. Quanto tempo a sua criança tem permissão para assistir a **cada dia da semana**?

Horas: Minutos:

1. Você limita a quantidade de tempo que a sua criança assiste TV, vídeos ou filmes **no final de semana** (sábado a domingo)?

🞎 Sim

🞎 Não →

**Se "NÃO", pule para a questão 27**

1. Quanto tempo a sua criança tem permissão para assistir a **cada dia do final de semana**?

Horas: Minutos:

1. Você limita a quantidade de tempo que a sua criança joga videogames **durante a semana** (segunda a sexta-feira)?

🞎 Sim

🞎 Não →

**Se "NÃO", pule para a questão 29**

1. Quanto tempo a sua criança tem permissão para jogar videogames a **cada dia da semana**?

Horas: Minutos:

1. Você limita a quantidade de tempo que a sua criança joga videogames **no final de semana** (sábado a domingo)?

🞎 Sim

🞎 Não →

**Se "NÃO", pule para a questão 31**

1. Quanto tempo a sua criança tem permissão para jogar videogames a **cada dia do final de semana**?

Horas: Minutos:

1. Marque um “X” no quadrado abaixo que melhor representa a frequência com que você faz cada uma das seguintes coisas.

| **Com que frequência você…** | **Nunca** | **Muito raramente** | **Raramente** | | **Às vezes** | | **Frequente** | | **Muito frequente** | |  |
| --- | --- | --- | --- | --- | --- | --- | --- | --- | --- | --- | --- |
| a. oferece TV, vídeo, ou filme a sua criança como recompensa por um bom comportamento? | 🞎 | 🞎 | | 🞎 | | 🞎 | | 🞎 | | 🞎 | |
| b. tira o tempo de TV, vídeo ou filme da sua criança como punição por um mal comportamento? | 🞎 | 🞎 | | 🞎 | | 🞎 | | 🞎 | | 🞎 | |
| c. oferece esportes ou atividades físicas a sua criança como recompensa por um bom comportamento? | 🞎 | 🞎 | | 🞎 | | 🞎 | | 🞎 | | 🞎 | |
| d. usa esportes ou atividades físicas para fazer com que a sua criança faça algo? (*por exemplo: “você não poderá brincar lá fora até almoçar”.*) | 🞎 | 🞎 | | 🞎 | | 🞎 | | 🞎 | | 🞎 | |

1. Marque um “X” no quadrado abaixo que melhor representa o quanto você concorda ou não concorda com cada uma das seguintes frases.

| **Eu monitoro de forma rigorosa o tempo que minha criança…** | **Não concordo totalmente** | **Não concordo** | **Não concordo e nem discordo** | **Concordo** | **Concordo totalmente** |
| --- | --- | --- | --- | --- | --- |
| a. assiste TV ou vídeos durante a **semana** (segunda a sexta-feira) | 🞎 | 🞎 | 🞎 | 🞎 | 🞎 |
| b. assiste TV ou vídeos durante o **final de semana** (sábado e domingo) | 🞎 | 🞎 | 🞎 | 🞎 | 🞎 |
| c. joga videogames durante a **semana** (segunda a sexta-feira) | 🞎 | 🞎 | 🞎 | 🞎 | 🞎 |
| d. joga videogames durante o **final de semana** (sábado e domingo) | 🞎 | 🞎 | 🞎 | 🞎 | 🞎 |

1. Quantos **dias por semana** sua família deixa a televisão ligada durante o **café da manhã**?

🞎 0 dias

🞎 1 dia

🞎 2 dias

🞎 3 dias

🞎 4 dias

🞎 5 dias

🞎 6 dias

🞎 7 dias

1. Quantos **dias por semana** sua família deixa a televisão ligada durante **o jantar**?

🞎 0 dias

🞎 1 dia

🞎 2 dias

🞎 3 dias

🞎 4 dias

🞎 5 dias

🞎 6 dias

🞎 7 dias

**35.** Marque um “X” no quadrado abaixo que melhor representa a frequência com que você faz cada uma das seguintes coisas.

| **Com que frequência…** | **Nunca** | **Muito raramente** | **Raramente** | **Às vezes** | **Frequente** | **Muito frequente** |
| --- | --- | --- | --- | --- | --- | --- |
| 1. **sua criança** ganha um tempo extra para assistir TV, vídeos ou filmes como recompensa? | 🞎 | 🞎 | 🞎 | 🞎 | 🞎 | 🞎 |
| 1. **sua criança** ganha um tempo extra para brincar fora de casa como recompensa? | 🞎 | 🞎 | 🞎 | 🞎 | 🞎 | 🞎 |
| 1. **você** usa o tempo de TV para controlar o comportamento da sua criança*?* *(por exemplo: “se você não parar com isto, não poderá assistir TV hoje.”)* | 🞎 | 🞎 | 🞎 | 🞎 | 🞎 | 🞎 |
| 1. **você** usa o esporte ou atividades físicas para controlar o comportamento da sua criança*? (por exemplo: “se você não parar com isto, não vai poder ir ao futebol/aula de dança hoje a tarde.”)* | 🞎 | 🞎 | 🞎 | 🞎 | 🞎 | 🞎 |
| 1. **você** diminue o tempo da sua criança de brincar do lado de fora de casa por mau comportamento? | 🞎 | 🞎 | 🞎 | 🞎 | 🞎 | 🞎 |

1. Marque um “X” no quadrado abaixo que melhor representa o quanto você concorda ou não concorda com cada uma das seguintes frases.

|  | **Não concordo totalmente** | **Não concordo** | **Não concordo e nem discordo** | **Concordo** | **Concordo totalmente** |
| --- | --- | --- | --- | --- | --- |
| 1. **Eu** tenho controle sobre o quanto de tempo de TV minha criança assiste. | 🞎 | 🞎 | 🞎 | 🞎 | 🞎 |
| 1. **Outros adultos** que convivem na vida da minha criança dificultam que ele/ela seja fisicamente ativo(a). | 🞎 | 🞎 | 🞎 | 🞎 | 🞎 |
| 1. Minha **família** é fisicamente ativa. | 🞎 | 🞎 | 🞎 | 🞎 | 🞎 |
| 1. **Eu** gosto de assistir TV/filmes com minha criança. | 🞎 | 🞎 | 🞎 | 🞎 | 🞎 |

1. O quanto **você** gosta de atividade física ou esporte?

🞎 Não gosto

🞎 Gosto um pouco

🞎 Gosto razoavelmente

🞎 Gosto muito

1. O quanto **você** gosta de assistir TV ou filmes durante o seu tempo livre?

🞎 Não gosto

🞎 Gosto um pouco

🞎 Gosto razoavelmente

🞎 Gosto muito

1. Com que frequência **sua família** usa a prática de atividades físicas ou esportes como uma forma de recreação familiar *(por exemplo, andar de bicicleta juntos, caminhar, jogar futebol/vôlei)?*

🞎 Raramente

🞎 De vez em quando

🞎 Com relativa frequência

🞎 Frequentemente

1. Com que frequência **você** vai assistir a **sua criança** em eventos esportivos, aulas ou competições organizadas de atividades físicas com ele/ela *(por exemplo, assistir a sua criança em competição de futebol ou vôlei, apresentação de dança, ou sessões de treinamento)*?

🞎 Raramente

🞎 De vez em quando

🞎 Com relativa frequência

🞎 Frequentemente

1. Quão importante é para **você** que a **sua criança** seja fisicamente ativa?

🞎 Não é importante

🞎 De pouco valor

🞎 Moderadamente importante

🞎 Importante

🞎 Muito importante

1. Durante o **ano passado**, algum adulto da sua família **pagou** para que a sua criança pudesse ter aulas ou praticar esportes que envolvessem atividade física moderada ou vigorosa *(por exemplo, dança, futebol, basquete, natação, ginástica)?*

🞎 Sim

🞎 Não →

**Se "NÃO", pule para a questão 44**

1. Para quantas atividades você ou outro adulto pagou?

**Atividades**

1. Com que frequência você usa **seu próprio comportamento** para incentivar a sua criança a ser fisicamente ativa?

| 🞎 Eu não uso meu próprio comportamento para incentivar minha criança a ser ativa. |
| --- |
| 🞎 Eu raramente uso meu próprio comportamento para incentivar minha criança a ser ativa. |
| 🞎 Eu frequentemente uso meu próprio comportamento para incentivar minha criança a ser ativa. |
| 🞎 Eu sempre uso meu próprio comportamento para incentivar minha criança a ser ativa. |

1. Com que frequência você matricula a sua criança em esportes?

| 🞎 Eu raramente matriculo minha criança em esportes. |
| --- |
| 🞎 De vez em quando eu matriculo minha criança em esportes. |
| 🞎 Eu frequentemente matriculo minha criança em esportes. |
| 🞎 Eu sempre matriculo minha criança em esportes. |

1. Durante o **último mês**, quantas vezes **você** levou a sua criança para brincar em um parque, praça, ou outra área de lazer da região?

**Vez(es) no último mês**

1. Marque um “X” no quadrado abaixo que melhor representa o quanto você concorda ou não concorda com cada uma das seguintes frases.

|  | | **Não concordo totalmente** | **Não concordo** | | | **Não concordo e nem discordo** | | | **Concordo** | | | **Concordo totalmente** |  |
| --- | --- | --- | --- | --- | --- | --- | --- | --- | --- | --- | --- | --- | --- |
| 1. **Eu** sou o responsável pela quantidade de TV que minha criança assiste durante seu tempo livre em casa. | | 🞎 | 🞎 | 🞎 | | | 🞎 | | | 🞎 | | |  |
| 1. **Eu** posso fazer com que minha criança seja fisicamente ativa em casa. | 🞎 | | 🞎 | | 🞎 | | | 🞎 | | | 🞎 | | |
| 1. **Outros adultos** que tem contato com a minha criança dificultam as regras de casa sobre o tempo permitido para ele/ela assistir televisão. | 🞎 | | 🞎 | | 🞎 | | | 🞎 | | | 🞎 | |  |
| 1. **Eu** gosto de ser fisicamente ativo(a) com minha criança. | 🞎 | | 🞎 | | 🞎 | | | 🞎 | | | 🞎 | |  |

1. Marque um “X” no quadrado abaixo que melhor representa a frequência com que você faz cada uma das seguintes coisas durante uma semana normal.

| **Durante uma semana normal, com que frequência...** | **Nunca** | **Muito raramente** | **Raramente** | **Algumas vezes** | **Frequente** | **Muito frequente** |
| --- | --- | --- | --- | --- | --- | --- |
| 1. **você** diz a sua criança como os hábitos sedentários *(por exemplo, ficar sentado por muito tempo)* podem ser prejudiciais para a saúde? | 🞎 | 🞎 | 🞎 | 🞎 | 🞎 | 🞎 |
| 1. **você** assiste TV ou vídeos com a sua criança? | 🞎 | 🞎 | 🞎 | 🞎 | 🞎 | 🞎 |
| 1. **você** manda a sua criança ir brincar fora de casa para que você possa fazer as atividades domésticas da sua casa? | 🞎 | 🞎 | 🞎 | 🞎 | 🞎 | 🞎 |
| 1. **você** leva a sua criança para brincar no parque? | 🞎 | 🞎 | 🞎 | 🞎 | 🞎 | 🞎 |

1. Marque um “X” no quadrado abaixo que melhor representa a frequência com que cada uma das seguintes coisas acontecem durante uma semana normal.

| **Durante uma semana normal, com que frequência...** | **Nunca** | **Muito raramente** | **Raramente** | **Algumas vezes** | **Frequente** | **Muito frequente** |
| --- | --- | --- | --- | --- | --- | --- |
| 1. **você** diz a sua criança que fazer atividade física é bom para a saúde? | 🞎 | 🞎 | 🞎 | 🞎 | 🞎 | 🞎 |
| 1. **seu** comportamento incentiva sua criança a ser sedentária*?* *(por exemplo, assistir TV por muito tempo.)* | 🞎 | 🞎 | 🞎 | 🞎 | 🞎 | 🞎 |
| 1. **você** elogia a sua criança por participar em esportes ou atividades físicas? | 🞎 | 🞎 | 🞎 | 🞎 | 🞎 | 🞎 |
| 1. **você** liga a TV, um vídeo ou um filme para sua criança assistir quando o tempo está ruim? *(por exemplo, quando está chovendo, muito quente ou muito frio.)* | 🞎 | 🞎 | 🞎 | 🞎 | 🞎 | 🞎 |
| 1. **você** diz coisas para incentivar a sua criança a fazer atividades físicas ou praticar esportes? | 🞎 | 🞎 | 🞎 | 🞎 | 🞎 | 🞎 |

1. Marque um “X” no quadrado abaixo que melhor representa a frequência com que cada uma das seguintes coisas acontecem durante uma semana normal.

| **Durante uma semana normal, com que frequência...** | **Nunca** | | **Muito raramente** | | **Raramente** | | **Algumas vezes** | | **Frequente** | | **Muito frequente** |
| --- | --- | --- | --- | --- | --- | --- | --- | --- | --- | --- | --- |
| 1. a sua criança **ouve você dizer** que estava cansado(a) demais para fazer atividades físicas? | | 🞎 | 🞎 | 🞎 | | 🞎 | | 🞎 | | 🞎 | |
| 1. a sua criança **vê** você assistindo TV ou filmes? | | 🞎 | 🞎 | 🞎 | | 🞎 | | 🞎 | | 🞎 | |
| 1. **você** pratica esportes, participa de jogos ativos ou faz outras atividades físicas com a sua criança? | | 🞎 | 🞎 | 🞎 | | 🞎 | | 🞎 | | 🞎 | |
| 1. **você** tenta incentivar a sua criança a brincar fora de casa quando o tempo está bom? | | 🞎 | 🞎 | 🞎 | | 🞎 | | 🞎 | | 🞎 | |
| 1. **você** leva a sua criança a algum lugar onde ele/ela possa ser fisicamente ativo(a) ou praticar esportes? | | 🞎 | 🞎 | 🞎 | | 🞎 | | 🞎 | | 🞎 | |

1. Marque um “X” no quadrado abaixo que melhor representa a frequência com que cada uma das seguintes coisas acontecem durante uma semana normal.

| **Durante uma semana normal, com que frequência...** | **Nunca** | | **Muito raramente** | | **Raramente** | | **Algumas vezes** | | **Frequente** | | | **Muito frequente** |  |  |
| --- | --- | --- | --- | --- | --- | --- | --- | --- | --- | --- | --- | --- | --- | --- |
| 1. a sua criança **ouve você** falar sobre participar de algum esporte ou ser fisicamente ativo(a)? | | | 🞎 | | 🞎 | | 🞎 | | 🞎 | | 🞎 | 🞎 | | |
| 1. a sua criança **vê** você fazendo ou indo fazer algo que seja fisicamente ativo *(por exemplo, caminhar, andar de bicicleta, praticar esportes)?* | | | 🞎 | | 🞎 | | 🞎 | | 🞎 | | 🞎 | 🞎 | | |
| 1. **você** liga a TV, um vídeo ou um filme para sua criança para que você possa fazer as atividades domésticas da sua casa? | | | 🞎 | | 🞎 | | 🞎 | | 🞎 | | 🞎 | 🞎 | | |
| 1. **você** tenta fazer com que a sua criança seja fisicamente ativa ao invés de assistir TV? | | | 🞎 | | 🞎 | | 🞎 | | 🞎 | | 🞎 | 🞎 | | |
| 1. **você** diz coisas para incentivar a sua criança a gastar menos tempo sendo sedentário*?* *(por exemplo, “pare de assistir e vá para rua brincar.”)* | | | 🞎 | | 🞎 | | 🞎 | | 🞎 | | 🞎 | 🞎 | | |

1. Marque um “X” no quadrado abaixo que melhor representa o quão importante cada uma das seguintes frases é para você.

| **O quão importante é para você que a sua criança...** | **Não é importante** | | **Um pouco importante** | | **Moderadamente importante** | | **Importante** | | **Muito importante** | |
| --- | --- | --- | --- | --- | --- | --- | --- | --- | --- | --- |
| 1. participe em esportes? | | 🞎 | | 🞎 | | 🞎 | | 🞎 | | 🞎 |
| 1. seja fisicamente ativa quando crescer? | | 🞎 | | 🞎 | | 🞎 | | 🞎 | | 🞎 |

| ***Sobre suas Interações com a sua Criança em Relação ao Sono*** |
| --- |

*As perguntas a seguir são sobre como você interage com a sua criança em relação ao sono.*

**Para responder as questões, lembre-se que:**

As rotinas de dormir são uma sequência definida de atividades que ocorrem geralmente na mesma ordem e com o mesmo adulto responsável pela criança antes dele/dela dormir. Marque um “X” no quadrado abaixo que melhor representa a rotina de dormir da sua criança no último mês.

| 1. **Durante as noites da *semana* do último mês, com que frequência a sua criança…** | | | | **Quase nunca** | | | **Algumas vezes** | | | **Metade das vezes** | | | | | **Frequente** | | | | | | | **Quase sempre** | | | | | | |
| --- | --- | --- | --- | --- | --- | --- | --- | --- | --- | --- | --- | --- | --- | --- | --- | --- | --- | --- | --- | --- | --- | --- | --- | --- | --- | --- | --- | --- |
| 1. realizou as **mesmas atividades** na hora antes de ir para a cama *(por exemplo, tomar banho, escovar os dentes, ler ou ouvir histórias, ouvir música)*? | | | | 🞎 | | | 🞎 | | | 🞎 | | | | | 🞎 | | | | | | | 🞎 | | | | | | |
| 1. realizou as atividades **na mesma ordem** antes de ir para a cama *(por exemplo, tomar banho, escovar os dentes, ler ou ouvir histórias, ouvir música)*? | | | | 🞎 | | | 🞎 | | | 🞎 | | | | | 🞎 | | | | | | | 🞎 | | | | | | |
| 1. dormiu **no mesmo lugar** *(por exemplo, na cama dele/dela, na cama dos pais, no sofá)*? | | | | 🞎 | | | 🞎 | | | 🞎 | | | | | 🞎 | | | | | | | 🞎 | | | | | | |
| 1. foi para a cama **no mesmo horário** (dentro de 10 minutos do horário previsto)? | | | | 🞎 | | | 🞎 | | | 🞎 | | | | | 🞎 | | | | | | | 🞎 | | | | | | |
| 1. foi colocado para dormir **pelo mesmo adulto responsável**? | | | | 🞎 | | | 🞎 | | | 🞎 | | | | | 🞎 | | | | | | | 🞎 | | | | | | |
|  | |  | | |  | | | |  | | |  | | | | | |  | | | | | |  |  |  |  |  |
| 1. **Durante as noites do *final de semana* do último mês, com que frequência a sua criança...** | | | | **Quase nunca** | | | **Algumas vezes** | | | **Metade das vezes** | | | | **Frequente** | | | | | | | **Quase sempre** | | | | |  |  |  |
| 1. realizou as **mesmas atividades** na hora antes de ir para a cama *(por exemplo, tomar banho, escovar os dentes, ler ou ouvir histórias, ouvir música)*? | | | | 🞎 | | | 🞎 | | | 🞎 | | | | 🞎 | | | | | | | 🞎 | | | | |  |  |  |
| 1. realizou as atividades **na mesma ordem** antes de ir para a cama *(por exemplo, tomar banho, escovar os dentes, ler ou ouvir histórias, ouvir música)*? | | | | 🞎 | | | 🞎 | | | 🞎 | | | | 🞎 | | | | | | | 🞎 | | | | |  |  |  |
| 1. dormiu **no mesmo lugar** *(por exemplo, na cama dele/dela, na cama dos pais, no sofá)*? | | | | 🞎 | | | 🞎 | | | 🞎 | | | | 🞎 | | | | | | | 🞎 | | | | |  |  |  |
| 1. foi para a cama **no mesmo horário** (dentro de 10 minutos do horário previsto)? | | | | 🞎 | | | 🞎 | | | 🞎 | | | | 🞎 | | | | | | | 🞎 | | | | |  |  |  |
| 1. foi colocado para dormir **pelo mesmo adulto responsável**? | | | | 🞎 | | | 🞎 | | | 🞎 | | | | 🞎 | | | | | | | 🞎 | | | | |  |  |  |
| 1. **Quão chateado a sua criança fica se ele/ela NÃO…** | | **Nenhum pouco** | | | **Um pouco** | | **Moderada-mente** | | | | | **Bastante** | | | | **Extrema-mente** | | | | | | | |  |  |  |  |  |
| 1. realiza as **mesmas atividades** na hora antes de ir para a cama (*por exemplo, tomar banho, escovar os dentes, ler ou ouvir histórias, ouvir música*)? | | 🞎 | | | 🞎 | | 🞎 | | | | | 🞎 | | | | 🞎 | | | | | | | |  |  |  |  |  |
| 1. realiza as atividades **na mesma ordem** antes de ir para a cama (*por exemplo, tomar banho, escovar os dentes, ler ou ouvir histórias, ouvir música*)? | | 🞎 | | | 🞎 | | 🞎 | | | | | 🞎 | | | | 🞎 | | | | | | | |  |  |  |  |  |
| 1. dorme **no mesmo lugar** (*por exemplo, na cama dele/dela, na cama dos pais, no sofá*)? | | 🞎 | | | 🞎 | | 🞎 | | | | | 🞎 | | | | 🞎 | | | | | | | |  |  |  |  |  |
| 1. vai para a cama **no mesmo horário** (dentro de 10 minutos do horário previsto)? | | 🞎 | | | 🞎 | | 🞎 | | | | | 🞎 | | | | 🞎 | | | | | | | |  |  |  |  |  |
| 1. for colocado para dormir **pelo mesmo adulto responsável**? | | 🞎 | | | 🞎 | | 🞎 | | | | | 🞎 | | | | 🞎 | | | | | | | |  |  |  |  |  |
|  | | | | | | | | | | | | | | | | | | | | | |  |  |  |  |  |  |  |
| 1. **No mês passado, na hora antes de ir para cama, com que frequência a sua criança…** | | **Quase nunca** | | | **Algumas vezes** | | | | | **A metade das vezes** | | | | | **Frequente** | | | | **Quase sempre** | | | | | | | |  |  |
| 1. Lêu/ouviu uma história? | | 🞎 | | | 🞎 | | | | | 🞎 | | | | | 🞎 | | | | 🞎 | | | | | | | |  |  |
| 1. Brincou com jogos ou brinquedos? | | 🞎 | | | 🞎 | | | | | 🞎 | | | | | 🞎 | | | | 🞎 | | | | | | | |  |  |
| 1. Brincou de forma ativa *(por exemplo, correu ou deu cambalhota)*? | | 🞎 | | | 🞎 | | | | | 🞎 | | | | | 🞎 | | | | 🞎 | | | | | | | |  |  |
| 1. Assitiu TV? | | 🞎 | | | 🞎 | | | | | 🞎 | | | | | 🞎 | | | | 🞎 | | | | | | | |  |  |
| 1. Jogou videogame? | | 🞎 | | | 🞎 | | | | | 🞎 | | | | | 🞎 | | | | 🞎 | | | | | | | |  |  |
| 1. Escutou música? | | 🞎 | | | 🞎 | | | | | 🞎 | | | | | 🞎 | | | | 🞎 | | | | | | | |  |  |
| 1. Comeu um lanche ou bebeu algo? | | 🞎 | | | 🞎 | | | | | 🞎 | | | | | 🞎 | | | | 🞎 | | | | | | | |  |  |
| 1. Tomou um banho? | | 🞎 | | | 🞎 | | | | | 🞎 | | | | | 🞎 | | | | 🞎 | | | | | | | |  |  |
| 1. Escovou os dentes? | | 🞎 | | | 🞎 | | | | | 🞎 | | | | | 🞎 | | | | 🞎 | | | | | | | |  |  |
| 1. Usou o banheiro (privada)? | | 🞎 | | | 🞎 | | | | | 🞎 | | | | 🞎 | | | | 🞎 | | | | | | | |  |  |  |
| 1. Abraçou/beijou o adulto responsável (*por exemplo, deu um beijo de boa noite*)? | | 🞎 | | | 🞎 | | | | | 🞎 | | | | 🞎 | | | | 🞎 | | | | | | | |  |  |  |
| 1. Disse boa noite aos membros da família? | | 🞎 | | | 🞎 | | | | | 🞎 | | | | 🞎 | | | | 🞎 | | | | | | | |  |  |  |
| 1. Foi colocado na cama? | | 🞎 | | | 🞎 | | | | | 🞎 | | | | 🞎 | | | | 🞎 | | | | | | | |  |  |  |
| 1. Colocou o pijama/roupa de dormir? | | 🞎 | | | 🞎 | | | | | 🞎 | | | | 🞎 | | | | 🞎 | | | | | | | |  |  |  |
| 1. Fez orações? | | 🞎 | | | 🞎 | | | | | 🞎 | | | | 🞎 | | | | 🞎 | | | | | | | |  |  |  |
| 1. Abraçou o adulto responsável *(por exemplo, a criança sentou no colo do responsável e ficou abraçado com ele/ela)*? | | 🞎 | | | 🞎 | | | | | 🞎 | | | | 🞎 | | | | 🞎 | | | | | | | |  |  |  |

**Você terminou!**

**Muito obrigada pelo seu tempo e esforço!**
