# Supplementary material for: Cross-Cultural Adaptation of Instruments Measuring Children’s Movement Behaviors and Parenting Practices in Brazilian Families
Source: Int J Environ Res Public Health. 2020 Dec 31;18(1):239. doi: 10.3390/ijerph18010239 (PMC7794996; doi:10.3390/ijerph18010239)
Supplement: Supplementary file 1 [file ijerph-18-00239-s001.zip › Supplementary File 3_With Scoring.docx]

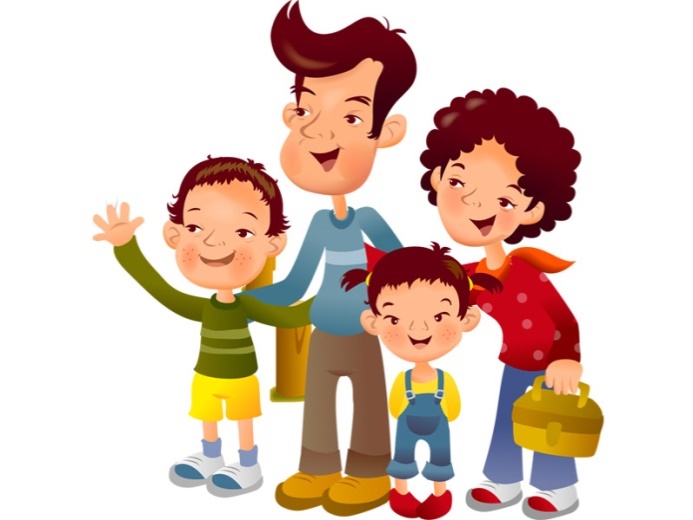


Family Health Survey

| - The purpose of this survey is to obtain information about your health behavior and the health behaviors of your child. - Please respond only about your oldest child aged 3 to 6 who is enrolled at Child Care/ Pre-school. - Be as accurate as you can – there are no right or wrong answers. - All information is strictly confidential. - Please try not to miss any questions and provide only one answer for each question. - If you are unsure on how to answer any question, please do not hesitate to ask the research team for help. - Your responses are important to us. - Answering this survey will take about 45 minutes of your time. |
| --- |

Identification

Child’s Name: _______________________________________________________________________

Child Care/Pre-school: ________________________________________________________________

Class: ( ) Berçario II ( ) Pré I ( ) Pré II

| ***Your Child’s Personal Information*** |
| --- |

Participant ID: __________

Child Care ID: __________

*This section of the survey is about your child.*

1. What is your child’s sex?

🞎 Female

🞎 Male

1. What is your child’s date of birth?

**_______ / _______ / _______**

day month year

1. What is your child ethinicity?

🞎 Caucasian

🞎 Mongoloid

🞎 Mixed-race

🞎 Afro Brazilians

🞎 Indigenous

1. What time does your child attend a day care center/pre-school?

🞎 Half-time (morning or afternoon)

🞎 Full-time (morning and afternoon)

| ***About You….*** |
| --- |

*This section of the survey is about you and your family.*

1. How old are you?

🞎 Up to 24 years old

🞎 Between 25 and 35 years old

🞎 Between 36 and 45 years old

🞎 Older than 46 years old

1. What is your relationship to the child?

🞎 Female adult caregiver (e.g. mother, mother-in-law, grandmother, aunt)

🞎 Male adult caregiver (e.g. father, father-in-law, grandfather, uncle)

1. What is your highest level of education?

🞎 No study

🞎 Incomplete Elementary School

🞎 Complete Elementary School

🞎 Incomplete High School

🞎 Complete High School

🞎 Incomplete Tertiary Education

🞎 Complete Tertiary Education

🞎 Post-graduation

1. What is your marital status?

🞎 Single

🞎 Married

🞎 Living with partner

🞎 Separated or divorced

🞎 Widowed

1. How would you describe your current employment status?

🞎 Employed full-time ‘full day’ (include self-employed)

🞎 Employed part-time (include self-employed)

🞎 Casually employed

🞎 Unable to work due to health problems

🞎 Unemployed

🞎 Retired

1. What was the income range of the family in the last month?

🞎 Up to R$998

🞎 Between R$998 and R$1497

🞎 Between R$1498 and R$1996

🞎 Greater than R$1997

1. Do you receive financial support from the government through the ‘Bolsa Família Programme’ because your child attends the Child Care/ Pre-school?

🞎 Yes

🞎 No

1. How many people live in your house? (Including you).

🞎 One

🞎 Two

🞎 Three

🞎 Four

🞎 Five or more

*The following questions are about your child’s weekly physical activity.*

Think a moment about a normal weekday for your child in the last month.

1. How much time would you say your child spends playing outdoors on a normal **weekday**?

Hours: Minutes:

Now think about a normal weekend day for your child in the last month*.*

1. How much time would you say your child spends playing outdoors on a normal **weekend day**?

Hours: Minutes:

| ***About your Child’s Screen Time*** |
| --- |

*The following questions are about your child’s screen time. This includes the use of television, iPads/computer tablets, smartphones, game consoles and computers.*

| ***About your Child’s Physical Activity*** |
| --- |

1. Thinking about the last month, how much time does your child spend doing each of the following activities at home? Please report separately for weekdays and weekend days.

|  | On a normal **weekday**  Monday - Friday hours/minutes | On a normal **weekend day**  Saturday & Sunday hours/minutes |
| --- | --- | --- |
| **A. Watching TV/DVDs** | **hrs**  **mins** | **hrs**  **mins** |
| **B. Using a computer** | **mins**  **hrs** | **hrs**  **mins** |
| **C. Playing with a video game system (e.g. Nintendo DS, Playstation, Xbox, etc.)** | **mins**  **hrs** | **mins**  **hrs** |
| **D. Using smartphones and iPads/Tablet Computer** | **mins**  **hrs** | **hrs**  **mins** |

| ***About your Child’s Sleep*** |
| --- |

*The following questions are related to your child’s sleeps routine.*

Thinking about the last month…

1. What time does your child usually fall asleep at night?

On a normal **weekday**: Hours: Minutes:

On a normal **weekend day**: Hours: Minutes:

1. What time does your child usually wake up in the morning to start the day?

On a normal **weekday**: Hours: Minutes:

On a normal **weekend day**: Hours: Minutes:

| ***About your Interactions with your Child in Relation to Physical Activity and Screen Time*** |
| --- |

*The following questions are about the ways you interact with your child in relation to physical activity and screen time.* We are interested in what you do and how you feel. Please read each question and select the best answer for you. Take your time and answer as accurately as possible.

***F7s_PPA1.6***

1. How **often** is the TV in your house on when people are at home?

🞎 Very Rarely

🞎 Rarely

🞎 Sometimes

🞎 Often

🞎 Very Often

🞎 Always

1. Place an “X” in the box below that best represents how often your child is allowed to do each of the following activities while playing **inside your house.**

|  | **Always** | **Sometimes** | **Never** |
| --- | --- | --- | --- |
| 1. hopping, skipping, or galloping   ***F1c_PPA2.1a*** | 🞎 | 🞎 | 🞎 |
| 1. running around | 🞎  ***F1c_PPA2.1b*** | 🞎 | 🞎 |
| 1. chasing   ***F1c_PPA2.1c*** | 🞎 | 🞎 | 🞎 |
| 1. rough housing or wrestling   ***F1c_PPA2.1d*** | 🞎 | 🞎 | 🞎 |
| 1. jumping from a height | 🞎  ***F1c_PPA2.1e*** | 🞎 | 🞎 |
| 1. flipping (somersault) or tumbling   ***F1c_PPA2.1f***  ***F1c_PPA2.1g***  ***F1c_PPA2.1h***  ***F1c_PPA2.1i***  ***F1c_PPA2.1j***  ***F1c_PPA2.1k*** | 🞎 | 🞎 | 🞎 |
| 1. climbing | 🞎 | 🞎 | 🞎 |
| 1. swinging or hanging | 🞎 | 🞎 | 🞎 |
| 1. balancing | 🞎 | 🞎 | 🞎 |
| 1. piling up pillows and jumping on them | 🞎 | 🞎 | 🞎 |
| 1. throwing, kicking, or bouncing a ball | 🞎 | 🞎 | 🞎 |

**20.** Place an “X” in the box below that best represents how much you agree or disagree with each of the following statements.

|  | **Strongly disagree** | **Disagree** | **Neither agree nor disagree** | **Agree** | **Strongly agree** |
| --- | --- | --- | --- | --- | --- |
| 1. When my child is **inside the house** their play should be calm and quiet. | 🞎  ***F1c_PPA2.2*** | 🞎 | 🞎 | 🞎 | 🞎 |
| 1. When **inside** **the house**, my child can use toys and equipment for physically active play (*for example, gross motor activities like running, jumping, hopping, or tumbling*). | 🞎  ***F1c_PPA2.3R*** | 🞎 | 🞎 | 🞎 | 🞎 |

1. How **often** do you ask your child to calm down their **indoor play**?

***F1c_PPA2.4***

🞎 Never

🞎 Very Rarely

🞎 Rarely

🞎 Sometimes

🞎 Often

🞎 Very Often

1. Place an “X” in the box below that best represents how often you do each of the following things related to your child’s **outdoor play**.

| **How often do you…** | **Never**  ***F2c_PPA2.5*** | **Very Rarely** | **Rarely** | **Sometimes** | **Often** | **Very Often** |
| --- | --- | --- | --- | --- | --- | --- |
| a. ask your child **not** to run when they are playing **outside?** | 🞎  ***F2c_PPA2.6*** | 🞎 | 🞎 | 🞎 | 🞎 | 🞎 |
| b. ask your child to try and stay clean when playing **outside**. | 🞎  ***F6c_PPA2.7*** | 🞎 | 🞎 | 🞎 | 🞎 | 🞎 |
| c. let your child play **outside** on hot days. | 🞎 | 🞎 | 🞎 | 🞎 | 🞎 | 🞎 |
| d. let your child play **outside** on cold days. | 🞎  ***F6c_PPA2.8*** | 🞎 | 🞎 | 🞎 | 🞎 | 🞎 |
| e. ask your child to calm down their **outdoor** play? | 🞎  ***F2c_PPA2.10*** | 🞎  ***F2c_PPA2.9*** | 🞎 | 🞎 | 🞎 | 🞎 |
| f. ask your child not to get their clothes dirty while he/she is playing **outside.** | 🞎 | 🞎 | 🞎 | 🞎 | 🞎 | 🞎 |

1. Do you limit the amount of time your child watches TV, videos, or movies **during the week** (Monday –Friday)?

***DV3c_PPA2.12***

🞎 Yes

🞎 No → **If no, skip to question 25**

1. About how much time is your child allowed to watch **each weekday**?

***F4c_PPA2.13R***

Hours: Minutes:

1. Do you limit the amount of time your child watches TV, videos, or movies **on the weekend** (Saturday - Sunday)?

***DV3c_PPA2.14***

🞎 Yes

🞎 No → **If no, skip to question 27**

***F4c_PPA2.15R***

1. About how much time is your child allowed to watch each **weekend day**?

Hours: Minutes:

***DV4c_PPA2.16***

1. Do you limit the amount of time your child plays video games **during the week** (Monday - Friday)?

🞎 Yes

🞎 No → **If no, skip to question 29**

***F4c_PPA2.17R***

1. About how much time is your child allowed to play video games **each weekday** (Monday - Friday)?

Hours: Minutes:

***DV4c_PPA2.18***

1. Do you limit the amount of time your child plays video games **on the weekend** (Saturday - Sunday)?

🞎 Yes

🞎 No → **If no, skip to question 31**

***F4c_PPA2.19R***

1. How much time is your child allowed to play video games **each weekend day?**

Hours: Minutes:

1. Place an “X” in the box below that best represents how often you do each of the following things.

| **How often do you…** | **Never** | **Very rarely** | **Rarely** | **Some- times** | **Often** | **Very often** |
| --- | --- | --- | --- | --- | --- | --- |
| a. offer TV, video, or movie time to your child as a reward for good behavior? | 🞎  ***F5c_PPA2.20*** | 🞎 | 🞎 | 🞎 | 🞎 | 🞎 |
| b. take away TV, video, or movie time as a punishment for bad behavior? | 🞎  ***F5c_PPA2.21*** | 🞎 | 🞎 | 🞎 | 🞎 | 🞎 |
| c. offer sports or physical activities to your child as a reward for good behavior? | 🞎  ***F3c_PPA2.22*** | 🞎 | 🞎 | 🞎 | 🞎 | 🞎 |
| d. use sports or physical activities to get your child to do something? (for *example: “You can’t go outside to play until you eat your lunch”.*) | 🞎  ***F3c_PPA2.23*** | 🞎 | 🞎 | 🞎 | 🞎 | 🞎. |

1. Place an “X” in the box below that best represents how much you agree or disagree with each of the following statements.

| **I tightly monitor the time my child…** | **Strongly disagree** | **Disagree** | **Neither agree nor disagree** | **Agree** | **Strongly agree** |
| --- | --- | --- | --- | --- | --- |
| a. watches TV or Videos during the **week** (Monday - Friday) | 🞎 | 🞎  ***F4c_PPA2.24*** | 🞎 | 🞎 | 🞎 |
| b. watches TV or Videos on the **weekend** (Saturday - Sunday) | 🞎 | 🞎  ***F4c_PPA2.25*** | 🞎 | 🞎 | 🞎 |
| c. plays video games during the **week** (Monday - Friday) | 🞎  ***F4c_PPA2.26*** | 🞎 | 🞎 | 🞎 | 🞎 |
| d. plays video games on the **weekend** (Saturday - Sunday) | 🞎  ***F4c_PPA2.27*** | 🞎 | 🞎 | 🞎 | 🞎 |

***F7s_PPA2.28***

1. How many **days per week** does your family have the television on during **breakfast**?

🞎 0 days

🞎 1 day

🞎 2 days

🞎 3 days

🞎 4 days

🞎 5 days

🞎 6 days

🞎 7 days

***F7s_PPA2.29***

1. How many **days per week** does your family have the television on during **evening meal**?

🞎 0 days

🞎 1 day

🞎 2 days

🞎 3 days

🞎 4 days

🞎 5 days

🞎 6 days

🞎 7 days

1. Place an “X” in the box below that best represents how often you do each of the following things.

| **How often…** | **Never** | **Very rarely** | **Rarely** | **Some- times** | **Often** | **Very often** |
| --- | --- | --- | --- | --- | --- | --- |
| 1. does **your child** get extra TV, video, or movie time as a reward? | 🞎  ***F5c_PPA2.30*** | 🞎 | 🞎 | 🞎 | 🞎 | 🞎 |
| 1. does **your child** get extra outside time as a reward? | 🞎  ***F3c_PPA2.31*** | 🞎 | 🞎 | 🞎 | 🞎 | 🞎 |
| 1. do **you** use TV time to control your child’s behavior? (*for* *example: “If you don’t stop that you will not be able to watch TV today.”*) | 🞎 | 🞎  ***F5c_PPA2.32*** | 🞎 | 🞎 | 🞎 | 🞎 |
| 1. do **you** use sports or physical activities to control your child’s behavior? (*for example: “If you don’t stop that you will not be able to go to soccer/dance class this afternoon.”*) | 🞎 | 🞎  ***F3c_PPA2.33*** | 🞎 | 🞎 | 🞎 | 🞎 |
| 1. do **you** take outside time away from your child for bad behavior? | 🞎  ***F3c_PPA2.34*** | 🞎 | 🞎 | 🞎 | 🞎 | 🞎 |

1. Place an “X” in the box below that best represents how much you agree or disagree with each of the following statements.

|  | **Strongly disagree** | **Disagree** | **Neither agree nor disagree** | **Agree** | **Strongly agree** |
| --- | --- | --- | --- | --- | --- |
| 1. **I** have control over how much TV my child watches. | 🞎 | 🞎  ***F4c_PPA3.3*** | 🞎 | 🞎 | 🞎 |
| 1. **Others adults** in my child’s life make it hard to get my child to be physically active. | 🞎 | 🞎  ***F6s_PPA3.4R*** | 🞎 | 🞎 | 🞎 |
| 1. **My family** is physically active. | 🞎  ***F1s_PPA3.6*** | 🞎  ***F8s_PPA3.7*** | 🞎 | 🞎 | 🞎 |
| 1. **I** enjoy watching TV/movies with my child. | 🞎 | 🞎 | 🞎 | 🞎 | 🞎 |

***F1s_PPA3.10***

1. How much do **you** enjoy physical activity or sport?

🞎 Don’t enjoy

🞎 Sort of enjoy

🞎 Really enjoy

🞎 Thoroughly enjoy

***F8s_PPA3.11***

1. How much do **you** enjoy watching TV or movies during your free time?

🞎 Don’t enjoy

🞎 Sort of enjoy

🞎 Really enjoy

🞎 Thoroughly enjoy

1. How often does **your family** use physical activities or sports as a form of family recreation? *(for example, going on bike rides together, hiking, and playing soccer/volleyball)*?

***F1s_PPA3.12***

🞎 Rarely

🞎 Once in a while

🞎 Relatively often

🞎 Frequently

1. How often do **you** go to **your child’s** sporting events, lessons, or other organized physical activities with them? *(for example, watch your child in a football or volleyball competition, perform in a dance recital, or training sessions)*?

***F3s_PPA3.13***

🞎 Rarely

🞎 Once in a while

🞎 Relatively often

🞎 Frequently

***F5s_PPA3.14***

1. How valuable is it to **you** that **your child** be physically active?

🞎 Not valuable

🞎 Of little value

🞎 Moderately

🞎 Valuable

🞎 Very valuable

1. During the **past year** has an adult in your family **paid fees** so your child could take lessons, classes, or play sports involving moderate or vigorous physical activity? *(for example, dance, soccer, basketball, swimming, gymnastics)*?

***PPA3.15***

🞎 Yes

🞎 No → **If no, skip to question 44**

***DV1F3s_PPA3.16_CAT***

1. For how many activities have you or other adults paid fees?

**Activities**

***F1s_PPA3.17***

1. How often do you use **your own behavior** to encourage your child to be physically active?

| 🞎 I don’t use my own behavior to encourage my child to be active. |
| --- |
| 🞎 I rarely use my own behavior to encourage my child to be active. |
| 🞎 I often use my own behavior to encourage my child to be active. |
| 🞎 I always use my own behavior to encourage my child to be active. |

1. How often do **you** enroll your child in sports?

***F3s_PPA3.19***

| 🞎 I rarely enroll my child in sports. |
| --- |
| 🞎 I enroll my child once in a while. |
| 🞎 I frequently enroll my child in sports. |
| 🞎 I always enroll my child in sports. |

1. During the **last month**, how many times have **you** taken your child to play at a park, playground or other leisure area of the region?

***DV4F4s_PPA3.20_CAT***

**Time(s) in last month**

1. Place an “X in the box below that best represents how much you agree or disagree with each of the following statements.

|  | **Strongly disagree** | **Disagree** | **Neither agree nor disagree** | **Agree** | **Strongly agree** |
| --- | --- | --- | --- | --- | --- |
| 1. **I** am in charge of how much TV my child watches during their free time at home. | 🞎  ***F4c_PPA3.22*** | 🞎 | 🞎 | 🞎 | 🞎 |
| 1. **I** can get my child to be physically active at home. | 🞎 | 🞎  ***F6s_PPA3.26*** | 🞎 | 🞎 | 🞎 |
| 1. **Others adults** who have contact with my child make it hard to enforce household rules about how much time s/he is allowed to watch TV. | 🞎 | 🞎  ***F6s_PPA3.27R*** | 🞎 | 🞎 | 🞎 |
| 1. **I** like being physically active with my child. | 🞎  ***F1s_PPA3.28*** | 🞎 | 🞎 | 🞎 | 🞎 |

1. Place an "X" in the box below that best represents how often you do the following things during a normal week.

| **During a normal week, how often...** | **Never** | **Very rarely** | **Rarely** | **Some- times** | **Often** | **Very often** |
| --- | --- | --- | --- | --- | --- | --- |
| 1. do **you** tell your child how sedentary habits (*for example, sitting for a long time)* can be harmful to health? | 🞎  ***F2s_PPA4.3*** | 🞎 | 🞎 | 🞎 | 🞎 | 🞎 |
| 1. do **yo**u watch TV or videos with your child? | 🞎 | 🞎  ***F8s_PPA4.4*** | 🞎 | 🞎 | 🞎 | 🞎 |
| 1. do **you** send your child outside to play so you can do household chores in your home? | 🞎 | 🞎  ***F2s_PPA4.5*** | 🞎 | 🞎 | 🞎 | 🞎 |
| 1. do **you** take your child to the park to play? | 🞎 | 🞎  ***F4s_PPA4.6*** | 🞎 | 🞎 | 🞎 | 🞎 |

1. Place an "X" in the box below that best represents how often the following things happen during a normal week.

| **During a normal week, how often...** | **Never** | **Very rarely** | **Rarely** | **Some- times** | **Often** | **Very often** |
| --- | --- | --- | --- | --- | --- | --- |
| 1. do **you** tell your child that physical activity is good for health? | 🞎  ***F2s_PPA4.10*** | 🞎 | 🞎 | 🞎 | 🞎 | 🞎 |
| 1. does **your** behavior encourage your child to be sedentary? (*for example, watching TV for a long time.*) | 🞎  ***F1s_PPA4.11R*** | 🞎 | 🞎 | 🞎 | 🞎 | 🞎 |
| 1. do **you** praise your child for participating in sports or physical activities? | 🞎 | 🞎  ***F2s_PPA4.12*** | 🞎 | 🞎 | 🞎 | 🞎 |
| 1. do **you** turn on the TV, a video, or movie for your child when the weather is bad? (*for example, when it is raining, too hot or too cold.)* | 🞎 | 🞎  ***F8s_PPA4.13*** | 🞎 | 🞎 | 🞎 | 🞎 |
| 1. do **you** say things to encourage your child to do physical activities or play sports? | 🞎 | 🞎  ***F2s_PPA4.14*** | 🞎 | 🞎 | 🞎 | 🞎 |

1. Place an "X" in the box below that best represents how often each of the following things happen during a normal week.

| **During a normal week, how often...** | **Never** | **Very rarely** | **Rarely** | **Some- times** | **Often** | **Very often** |
| --- | --- | --- | --- | --- | --- | --- |
| 1. does your child **hear you say** that you were too tired to do physical activities? | 🞎 | 🞎  ***F1s_PPA4.16R*** | 🞎 | 🞎 | 🞎 | 🞎 |
| 1. does your child **see** **you** watching TV or movies? | 🞎 | 🞎  ***F8s_PPA4.17*** | 🞎 | 🞎 | 🞎 | 🞎 |
| 1. do **you** play sports, active games, or do other physical activities with your child? | 🞎 | 🞎  ***F1s_PPA4.18*** | 🞎 | 🞎 | 🞎 | 🞎 |
| 1. do **you** try to get your child to play outside when the weather is nice? | 🞎 | 🞎 | 🞎 | 🞎 | 🞎 | 🞎 |
| 1. do **you** transport your child to a place where they can be physically active or play sports? | 🞎 | 🞎  ***F4s_PPA4.20*** | 🞎 | 🞎 | 🞎 | 🞎 |

***F4s_PPA4.19***

1. Place an "X" in the box below that best represents how often each of the following things happen during a normal week.

| **During a normal week, how often...** | **Never** | **Very rarely** | **Rarely** | **Some- times** | **Often** | **Very often** |
| --- | --- | --- | --- | --- | --- | --- |
| 1. does your child **hear you** talk about participating in a sport or being physically active? | 🞎 | 🞎  ***F1s_PPA4.22*** | 🞎 | 🞎 | 🞎 | 🞎 |
| 1. does your child **see you** doing, or going to do, something that is physically active (*for example, walking, biking, playing sports*)? | 🞎  ***F1s_PPA4.23*** | 🞎 | 🞎 | 🞎 | 🞎 | 🞎 |
| 1. do **you** turn on the TV, a video, or movie for your child so you can do household chores in your home? | 🞎  ***F8s_PPA4.24*** | 🞎 | 🞎 | 🞎 | 🞎 | 🞎 |
| 1. do **you** try to get your child to be physically active instead of watching TV? | 🞎 | 🞎  ***F2s_PPA4.25*** | 🞎 | 🞎 | 🞎 | 🞎 |
| 1. do **you** say things to encourage your child to spend less time being sedentary? (*for example, "Stop watching TV and go outside and play".)* | 🞎 | 🞎  ***F2s_PPA4.26*** | 🞎 | 🞎 | 🞎 | 🞎 |

1. Place an "X" in the box below that best represents how important each of the following statements is to you.

| **How important is it for you that your child…** | **Unimportant** | **Of little importance** | **moderately important** | **important** | **very important** |
| --- | --- | --- | --- | --- | --- |
| 1. participate in sports? | 🞎 | 🞎 | 🞎 | 🞎 | 🞎 |
| 1. be physically active when they grow up?   ***F5s_PPA4.28***  ***F5s_PPA4.27*** | 🞎 | 🞎 | 🞎 | 🞎 | 🞎 |

| ***About your Interactions with your Child in Relation to Sleep*** |
| --- |

The following questions are about the ways you interact with your child in relation to sleep.

**To answer the questions remember that:**

Bedtime routines are a set sequence of activities that occur regularly in the same order and with the same caretaker before a child goes to bed. Place an "X" in the box that best represents your child's sleep routine in the last month.

| 1. **During *weeknights* for the past month, how often did your child…** | **Almost**  **never** | **Sometimes** | **Half the time** | **Often** | **Nearly**  **always** |
| --- | --- | --- | --- | --- | --- |
| 1. perform the **same activities** in the hour before going to bed *(for example, bathe, brush teeth, read/listen to story, listen to music)*? | 🞎 | 🞎  ***F1rb_BRQ1.1*** | 🞎 | 🞎 | 🞎 |
| 1. perform activities **in the same order** before going to bed *(for example, bathe, brush teeth, read/listen to story, listen to music)*? | 🞎  ***F1rb_BRQ1.2***  ***F2re_BRQ1.3*** | 🞎 | 🞎 | 🞎 | 🞎 |
| 1. sleep **in the same place** *(for example, in his/her bed, in parent’s bed, on couch)*? | 🞎 | 🞎 | 🞎 | 🞎 | 🞎 |
| 1. go to bed **at the same time** (within 10 minutes of the scheduled time)? | 🞎  ***F2re_BRQ1.4*** | 🞎 | 🞎 | 🞎 | 🞎 |
| 1. get put to bed **by the same person**? | 🞎 | 🞎  ***F2re_BRQ1.5*** | 🞎 | 🞎 | 🞎 |

| 1. **During *weekend nights* for the past month, how often did your child…** | | **Almost**  **never** | | **Sometimes** | | | **Half the time** | | | | **Often** | | | | **Nearly**  **always** |  |  |
| --- | --- | --- | --- | --- | --- | --- | --- | --- | --- | --- | --- | --- | --- | --- | --- | --- | --- |
| 1. perform the **same activities** in the hour before going to bed *(for example, bathe, brush teeth, read/listen to story, listen to music)*? | | 🞎 | | 🞎  ***F1rb_BRQ1.6*** | | | 🞎 | | | | 🞎 | | | | 🞎 |  |  |
| 1. perform activities **in the same order** before going to bed *(for example, bathe, brush teeth, read/listen to story, listen to music)*? | | 🞎 | | 🞎  ***F1rb_BRQ1.7*** | | | 🞎 | | | | 🞎 | | | | 🞎 |  |  |
| 1. sleep **in the same place** *(for example, in his/her bed, in parent’s bed, on couch)*? | | 🞎 | | 🞎  ***F2re_BRQ1.8*** | | | 🞎 | | | | 🞎 | | | | 🞎 |  |  |
| 1. go to bed **at the same time** (within 10 minutes of the scheduled time)? | | 🞎 | | 🞎  ***F2re_BRQ1.9*** | | | 🞎 | | | | 🞎 | | | | 🞎 |  |  |
| 1. get put to bed **by the same person**? | | 🞎 | | 🞎  ***F2re_BRQ1.10*** | | | 🞎 | | | | 🞎 | | | | 🞎 |  |  |
| 1. **How upset does your child get if he or she does NOT…** | **Not at all** | | **A little** | | **Moderately** | | | **Quite a bit** | | | | **Extremely** | | | |  |  |
| 1. perform the **same activities** in the hour before going to bed *(for example, bathe, brush teeth, read/listen to story, listen to music)*? | 🞎 | | 🞎  ***F3rt_BRQ1.11*** | | 🞎 | | | 🞎 | | | | 🞎 | | | |  |  |
| 1. perform activities **in the same order** before going to bed (*for example, bathe, brush teeth, read/listen to story, listen to music*)? | 🞎 | | 🞎  ***F3rt_BRQ1.12*** | | 🞎 | | | 🞎 | | | | 🞎 | | | |  |  |
| 1. sleep **in the same place** *(for example, in his/her bed, in parent’s bed, on couch*)? | 🞎 | | 🞎  ***F3rt_BRQ1.13*** | | 🞎 | | | 🞎 | | | | 🞎 | | | |  |  |
| 1. go to bed **at the same time** (within 10 minutes of the scheduled time)? | 🞎  ***F3rt_BRQ1.14*** | | 🞎 | | 🞎 | | | 🞎 | | | | 🞎 | | | |  |  |
| 1. get put to bed **by the same person**? | 🞎 | | 🞎  ***F3rt_BRQ1.15*** | | 🞎 | | | 🞎 | | | | 🞎 | | | |  |  |
|  | | | | | | | | | | | | | | | |  |  |
| 1. **In the past month, in the hour before going to bed, how often did your child…** | **Almost never** | | **Sometimes** | | | **Half the time** | | | | **Often** | | | | **Nearly always** | | | |
| 1. Read/listen to a story? | 🞎 | | 🞎  ***F4ac_BRQ1.16*** | | | 🞎 | | | | 🞎 | | | | 🞎 | | | |
| 1. Play with games or toys? | 🞎 | | 🞎  ***F5ma_BRQ1.17*** | | | 🞎 | | | | 🞎 | | | | 🞎 | | | |
| 1. Have active play (such as roughhouse or run around? | 🞎 | | 🞎  ***F5ma_BRQ1.18*** | | | 🞎 | | | | 🞎 | | | | 🞎 | | | |
| 1. Watch TV? | 🞎 | | 🞎  ***F5ma_BRQ1.19*** | | | 🞎 | | | | 🞎 | | | | 🞎 | | | |
| 1. Play video games? | 🞎 | | 🞎  ***F5ma_BRQ1.20*** | | | 🞎 | | | | 🞎 | | | | 🞎 | | | |
| 1. Listen to music? | 🞎 | | 🞎  ***F5ma_BRQ1.21*** | | | 🞎 | | | | 🞎 | | | | 🞎 | | | |
| 1. Have a snack or drink? | 🞎 | | 🞎  ***F5ma_BRQ1.22*** | | | 🞎 | | | | 🞎 | | | | 🞎 | | | |
| 1. Take a shower/bath? | 🞎 | | 🞎  ***F4ac_BRQ1.23*** | | | 🞎 | | | | 🞎 | | | | 🞎 | | | |
| 1. Brush teeth? | 🞎 | | 🞎  ***F4ac_BRQ1.24*** | | | 🞎 | | | | 🞎 | | | | 🞎 | | | |
| 1. Use the toilet? | 🞎  ***F4ac_BRQ1.26*** | | 🞎  ***F4ac_BRQ1.25*** | | | 🞎 | | | 🞎 | | | | 🞎 | | | |  |
| 1. Hug/kiss caregiver (*for example, a good night kiss*)? | 🞎  ***F4ac_BRQ1.27*** | | 🞎 | | | 🞎 | | | 🞎 | | | | 🞎 | | | |  |
| 1. Say goodnight to family members? | 🞎  ***F4ac_BRQ1.28*** | | 🞎 | | | 🞎 | | | 🞎 | | | | 🞎 | | | |  |
| 1. Get tucked in? | 🞎 | | 🞎 | | | 🞎 | | | 🞎 | | | | 🞎 | | | |  |
| 1. Put on pajamas? | 🞎 | | 🞎  ***F4ac_BRQ1.29*** | | | 🞎 | | | 🞎 | | | | 🞎 | | | |  |
| 1. Say prayers? | 🞎  ***F4ac_BRQ1.30*** | | 🞎 | | | 🞎 | | | 🞎 | | | | 🞎 | | | |  |
| 1. Cuddle with caregiver? (*for example, the child sat on the lap of the caregiver and was hugged with him/her)* | 🞎 | | 🞎  ***F4ac_BRQ1.31*** | | | 🞎 | | | 🞎 | | | | 🞎 | | | |  |

**You’re finished!**

**Thank you for your time and effort!**
