# Supplementary material for: Cross-Cultural Adaptation of Instruments Measuring Children’s Movement Behaviors and Parenting Practices in Brazilian Families
Source: Int J Environ Res Public Health. 2020 Dec 31;18(1):239. doi: 10.3390/ijerph18010239 (PMC7794996; doi:10.3390/ijerph18010239)
Supplement: Supplementary file 1 [file ijerph-18-00239-s001.zip › Supplementary File 3_Without Scoring.docx]

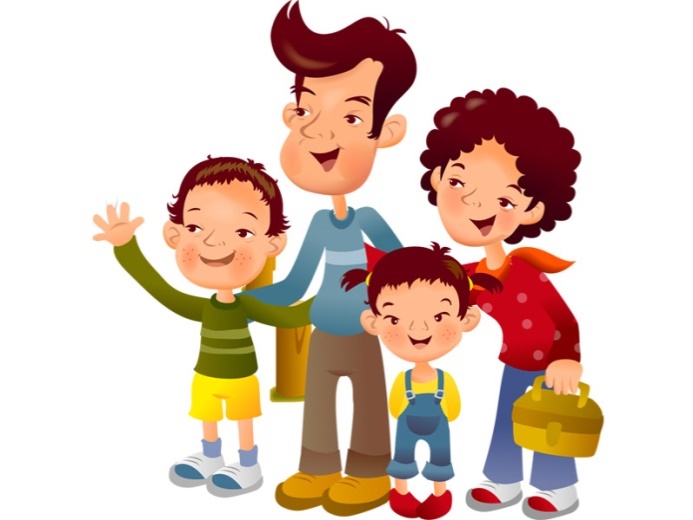


Family Health Survey

| - The purpose of this survey is to obtain information about your health behavior and the health behaviors of your child. - Please respond only about your oldest child aged 3 to 6 who is enrolled at Child Care/ Pre-school. - Be as accurate as you can – there are no right or wrong answers. - All information is strictly confidential. - Please try not to miss any questions and provide only one answer for each question. - If you are unsure on how to answer any question, please do not hesitate to ask the research team for help. - Your responses are important to us. - Answering this survey will take about 45 minutes of your time. |
| --- |

Identification

Child’s Name: _______________________________________________________________________

Child Care/Pre-school: ________________________________________________________________

Class: ( ) Berçario II ( ) Pré I ( ) Pré II

| ***Your Child’s Personal Information*** |
| --- |

Participant ID: __________

Child Care ID: __________

*This section of the survey is about your child.*

1. What is your child’s sex?

🞎 Female

🞎 Male

1. What is your child’s date of birth?

**_______ / _______ / _______**

day month year

1. What is your child ethinicity?

🞎 Caucasian

🞎 Mongoloid

🞎 Mixed-race

🞎 Afro Brazilians

🞎 Indigenous

1. What time does your child attend a day care center/pre-school?

🞎 Half-time (morning or afternoon)

🞎 Full-time (morning and afternoon)

| ***About You….*** |
| --- |

*This section of the survey is about you and your family.*

1. How old are you?

🞎 Up to 24 years old

🞎 Between 25 and 35 years old

🞎 Between 36 and 45 years old

🞎 Older than 46 years old

1. What is your relationship to the child?

🞎 Female adult caregiver (e.g. mother, mother-in-law, grandmother, aunt)

🞎 Male adult caregiver (e.g. father, father-in-law, grandfather, uncle)

1. What is your highest level of education?

🞎 No study

🞎 Incomplete Elementary School

🞎 Complete Elementary School

🞎 Incomplete High School

🞎 Complete High School

🞎 Incomplete Tertiary Education

🞎 Complete Tertiary Education

🞎 Post-graduation

1. What is your marital status?

🞎 Single

🞎 Married

🞎 Living with partner

🞎 Separated or divorced

🞎 Widowed

1. How would you describe your current employment status?

🞎 Employed full-time ‘full day’ (include self-employed)

🞎 Employed part-time (include self-employed)

🞎 Casually employed

🞎 Unable to work due to health problems

🞎 Unemployed

🞎 Retired

1. What was the income range of the family in the last month?

🞎 Up to R$998

🞎 Between R$998 and R$1497

🞎 Between R$1498 and R$1996

🞎 Greater than R$1997

1. Do you receive financial support from the government through the ‘Bolsa Família Programme’ because your child attends the Child Care/ Pre-school?

🞎 Yes

🞎 No

1. How many people live in your house? (Including you).

🞎 One

🞎 Two

🞎 Three

🞎 Four

🞎 Five or more

*The following questions are about your child’s weekly physical activity.*

Think a moment about a normal weekday for your child in the last month.

1. How much time would you say your child spends playing outdoors on a normal **weekday**?

Hours: Minutes:

Now think about a normal weekend day for your child in the last month*.*

1. How much time would you say your child spends playing outdoors on a normal **weekend day**?

Hours: Minutes:

| ***About your Child’s Screen Time*** |
| --- |

*The following questions are about your child’s screen time. This includes the use of television, iPads/computer tablets, smartphones, game consoles and computers.*

| ***About your Child’s Physical Activity*** |
| --- |

1. Thinking about the last month, how much time does your child spend doing each of the following activities at home? Please report separately for weekdays and weekend days.

|  | On a normal **weekday**  Monday - Friday hours/minutes | On a normal **weekend day**  Saturday & Sunday hours/minutes |
| --- | --- | --- |
| **A. Watching TV/DVDs** | **hrs**  **mins** | **hrs**  **mins** |
| **B. Using a computer** | **mins**  **hrs** | **hrs**  **mins** |
| **C. Playing with a video game system (e.g. Nintendo DS, Playstation, Xbox, etc.)** | **mins**  **hrs** | **mins**  **hrs** |
| **D. Using smartphones and iPads/Tablet Computer** | **mins**  **hrs** | **hrs**  **mins** |

| ***About your Child’s Sleep*** |
| --- |

*The following questions are related to your child’s sleeps routine.*

Thinking about the last month…

1. What time does your child usually fall asleep at night?

On a normal **weekday**: Hours: Minutes:

On a normal **weekend day**: Hours: Minutes:

1. What time does your child usually wake up in the morning to start the day?

On a normal **weekday**: Hours: Minutes:

On a normal **weekend day**: Hours: Minutes:

| ***About your Interactions with your Child in Relation to Physical Activity and Screen Time*** |
| --- |

*The following questions are about the ways you interact with your child in relation to physical activity and screen time.* We are interested in what you do and how you feel. Please read each question and select the best answer for you. Take your time and answer as accurately as possible.

1. How **often** is the TV in your house on when people are at home?

🞎 Very Rarely

🞎 Rarely

🞎 Sometimes

🞎 Often

🞎 Very Often

🞎 Always

1. Place an “X” in the box below that best represents how often your child is allowed to do each of the following activities while playing **inside your house.**

|  | **Always** | **Sometimes** | **Never** |
| --- | --- | --- | --- |
| 1. hopping, skipping, or galloping | 🞎 | 🞎 | 🞎 |
| 1. running around | 🞎 | 🞎 | 🞎 |
| 1. chasing | 🞎 | 🞎 | 🞎 |
| 1. rough housing or wrestling | 🞎 | 🞎 | 🞎 |
| 1. jumping from a height | 🞎 | 🞎 | 🞎 |
| 1. flipping (somersault) or tumbling | 🞎 | 🞎 | 🞎 |
| 1. climbing | 🞎 | 🞎 | 🞎 |
| 1. swinging or hanging | 🞎 | 🞎 | 🞎 |
| 1. balancing | 🞎 | 🞎 | 🞎 |
| 1. piling up pillows and jumping on them | 🞎 | 🞎 | 🞎 |
| 1. throwing, kicking, or bouncing a ball | 🞎 | 🞎 | 🞎 |

**20.** Place an “X” in the box below that best represents how much you agree or disagree with each of the following statements.

|  | **Strongly disagree** | **Disagree** | **Neither agree nor disagree** | **Agree** | **Strongly agree** |
| --- | --- | --- | --- | --- | --- |
| 1. When my child is **inside the house** their play should be calm and quiet. | 🞎 | 🞎 | 🞎 | 🞎 | 🞎 |
| 1. When **inside** **the house**, my child can use toys and equipment for physically active play (*for example, gross motor activities like running, jumping, hopping, or tumbling*). | 🞎 | 🞎 | 🞎 | 🞎 | 🞎 |

1. How **often** do you ask your child to calm down their **indoor play**?

🞎 Never

🞎 Very Rarely

🞎 Rarely

🞎 Sometimes

🞎 Often

🞎 Very Often

1. Place an “X” in the box below that best represents how often you do each of the following things related to your child’s **outdoor play**.

| **How often do you…** | **Never** | **Very Rarely** | **Rarely** | **Sometimes** | **Often** | **Very Often** |
| --- | --- | --- | --- | --- | --- | --- |
| a. ask your child **not** to run when they are playing **outside?** | 🞎 | 🞎 | 🞎 | 🞎 | 🞎 | 🞎 |
| b. ask your child to try and stay clean when playing **outside**. | 🞎 | 🞎 | 🞎 | 🞎 | 🞎 | 🞎 |
| c. let your child play **outside** on hot days. | 🞎 | 🞎 | 🞎 | 🞎 | 🞎 | 🞎 |
| d. let your child play **outside** on cold days. | 🞎 | 🞎 | 🞎 | 🞎 | 🞎 | 🞎 |
| e. ask your child to calm down their **outdoor** play? | 🞎 | 🞎 | 🞎 | 🞎 | 🞎 | 🞎 |
| f. ask your child not to get their clothes dirty while he/she is playing **outside.** | 🞎 | 🞎 | 🞎 | 🞎 | 🞎 | 🞎 |

1. Do you limit the amount of time your child watches TV, videos, or movies **during the week** (Monday –Friday)?

🞎 Yes

🞎 No → **If no, skip to question 25**

1. About how much time is your child allowed to watch **each weekday**?

Hours: Minutes:

1. Do you limit the amount of time your child watches TV, videos, or movies **on the weekend** (Saturday - Sunday)?

🞎 Yes

🞎 No → **If no, skip to question 27**

1. About how much time is your child allowed to watch each **weekend day**?

Hours: Minutes:

1. Do you limit the amount of time your child plays video games **during the week** (Monday - Friday)?

🞎 Yes

🞎 No → **If no, skip to question 29**

1. About how much time is your child allowed to play video games **each weekday** (Monday - Friday)?

Hours: Minutes:

1. Do you limit the amount of time your child plays video games **on the weekend** (Saturday - Sunday)?

🞎 Yes

🞎 No → **If no, skip to question 31**

1. How much time is your child allowed to play video games **each weekend day?**

Hours: Minutes:

1. Place an “X” in the box below that best represents how often you do each of the following things.

| **How often do you…** | **Never** | **Very rarely** | **Rarely** | **Some- times** | **Often** | **Very often** |
| --- | --- | --- | --- | --- | --- | --- |
| a. offer TV, video, or movie time to your child as a reward for good behavior? | 🞎 | 🞎 | 🞎 | 🞎 | 🞎 | 🞎 |
| b. take away TV, video, or movie time as a punishment for bad behavior? | 🞎 | 🞎 | 🞎 | 🞎 | 🞎 | 🞎 |
| c. offer sports or physical activities to your child as a reward for good behavior? | 🞎 | 🞎 | 🞎 | 🞎 | 🞎 | 🞎 |
| d. use sports or physical activities to get your child to do something? (for *example: “You can’t go outside to play until you eat your lunch”.*) | 🞎 | 🞎 | 🞎 | 🞎 | 🞎 | 🞎. |

1. Place an “X” in the box below that best represents how much you agree or disagree with each of the following statements.

| **I tightly monitor the time my child…** | **Strongly disagree** | **Disagree** | **Neither agree nor disagree** | **Agree** | **Strongly agree** |
| --- | --- | --- | --- | --- | --- |
| a. watches TV or Videos during the **week** (Monday - Friday) | 🞎 | 🞎 | 🞎 | 🞎 | 🞎 |
| b. watches TV or Videos on the **weekend** (Saturday - Sunday) | 🞎 | 🞎 | 🞎 | 🞎 | 🞎 |
| c. plays video games during the **week** (Monday - Friday) | 🞎 | 🞎 | 🞎 | 🞎 | 🞎 |
| d. plays video games on the **weekend** (Saturday - Sunday) | 🞎 | 🞎 | 🞎 | 🞎 | 🞎 |

1. How many **days per week** does your family have the television on during **breakfast**?

🞎 0 days

🞎 1 day

🞎 2 days

🞎 3 days

🞎 4 days

🞎 5 days

🞎 6 days

🞎 7 days

1. How many **days per week** does your family have the television on during **evening meal**?

🞎 0 days

🞎 1 day

🞎 2 days

🞎 3 days

🞎 4 days

🞎 5 days

🞎 6 days

🞎 7 days

1. Place an “X” in the box below that best represents how often you do each of the following things.

| **How often…** | **Never** | **Very rarely** | **Rarely** | **Some- times** | **Often** | **Very often** |
| --- | --- | --- | --- | --- | --- | --- |
| 1. does **your child** get extra TV, video, or movie time as a reward? | 🞎 | 🞎 | 🞎 | 🞎 | 🞎 | 🞎 |
| 1. does **your child** get extra outside time as a reward? | 🞎 | 🞎 | 🞎 | 🞎 | 🞎 | 🞎 |
| 1. do **you** use TV time to control your child’s behavior? (*for* *example: “If you don’t stop that you will not be able to watch TV today.”*) | 🞎 | 🞎 | 🞎 | 🞎 | 🞎 | 🞎 |
| 1. do **you** use sports or physical activities to control your child’s behavior? (*for example: “If you don’t stop that you will not be able to go to soccer/dance class this afternoon.”*) | 🞎 | 🞎 | 🞎 | 🞎 | 🞎 | 🞎 |
| 1. do **you** take outside time away from your child for bad behavior? | 🞎 | 🞎 | 🞎 | 🞎 | 🞎 | 🞎 |

1. Place an “X” in the box below that best represents how much you agree or disagree with each of the following statements.

|  | **Strongly disagree** | **Disagree** | **Neither agree nor disagree** | **Agree** | **Strongly agree** |
| --- | --- | --- | --- | --- | --- |
| 1. **I** have control over how much TV my child watches. | 🞎 | 🞎 | 🞎 | 🞎 | 🞎 |
| 1. **Others adults** in my child’s life make it hard to get my child to be physically active. | 🞎 | 🞎 | 🞎 | 🞎 | 🞎 |
| 1. **My family** is physically active. | 🞎 | 🞎 | 🞎 | 🞎 | 🞎 |
| 1. **I** enjoy watching TV/movies with my child. | 🞎 | 🞎 | 🞎 | 🞎 | 🞎 |

1. How much do **you** enjoy physical activity or sport?

🞎 Don’t enjoy

🞎 Sort of enjoy

🞎 Really enjoy

🞎 Thoroughly enjoy

1. How much do **you** enjoy watching TV or movies during your free time?

🞎 Don’t enjoy

🞎 Sort of enjoy

🞎 Really enjoy

🞎 Thoroughly enjoy

1. How often does **your family** use physical activities or sports as a form of family recreation? *(for example, going on bike rides together, hiking, and playing soccer/volleyball)*?

🞎 Rarely

🞎 Once in a while

🞎 Relatively often

🞎 Frequently

1. How often do **you** go to **your child’s** sporting events, lessons, or other organized physical activities with them? *(for example, watch your child in a football or volleyball competition, perform in a dance recital, or training sessions)*?

🞎 Rarely

🞎 Once in a while

🞎 Relatively often

🞎 Frequently

1. How valuable is it to **you** that **your child** be physically active?

🞎 Not valuable

🞎 Of little value

🞎 Moderately

🞎 Valuable

🞎 Very valuable

1. During the **past year** has an adult in your family **paid fees** so your child could take lessons, classes, or play sports involving moderate or vigorous physical activity? *(for example, dance, soccer, basketball, swimming, gymnastics)*?

🞎 Yes

🞎 No → **If no, skip to question 44**

1. For how many activities have you or other adults paid fees?

**Activities**

1. How often do you use **your own behavior** to encourage your child to be physically active?

| 🞎 I don’t use my own behavior to encourage my child to be active. |
| --- |
| 🞎 I rarely use my own behavior to encourage my child to be active. |
| 🞎 I often use my own behavior to encourage my child to be active. |
| 🞎 I always use my own behavior to encourage my child to be active. |

1. How often do **you** enroll your child in sports?

| 🞎 I rarely enroll my child in sports. |
| --- |
| 🞎 I enroll my child once in a while. |
| 🞎 I frequently enroll my child in sports. |
| 🞎 I always enroll my child in sports. |

1. During the **last month**, how many times have **you** taken your child to play at a park, playground or other leisure area of the region?

**Time(s) in last month**

1. Place an “X in the box below that best represents how much you agree or disagree with each of the following statements.

|  | **Strongly disagree** | **Disagree** | **Neither agree nor disagree** | **Agree** | **Strongly agree** |
| --- | --- | --- | --- | --- | --- |
| 1. **I** am in charge of how much TV my child watches during their free time at home. | 🞎 | 🞎 | 🞎 | 🞎 | 🞎 |
| 1. **I** can get my child to be physically active at home. | 🞎 | 🞎 | 🞎 | 🞎 | 🞎 |
| 1. **Others adults** who have contact with my child make it hard to enforce household rules about how much time s/he is allowed to watch TV. | 🞎 | 🞎 | 🞎 | 🞎 | 🞎 |
| 1. **I** like being physically active with my child. | 🞎 | 🞎 | 🞎 | 🞎 | 🞎 |

1. Place an "X" in the box below that best represents how often you do the following things during a normal week.

| **During a normal week, how often...** | **Never** | **Very rarely** | **Rarely** | **Some- times** | **Often** | **Very often** |
| --- | --- | --- | --- | --- | --- | --- |
| 1. do **you** tell your child how sedentary habits (*for example, sitting for a long time)* can be harmful to health? | 🞎 | 🞎 | 🞎 | 🞎 | 🞎 | 🞎 |
| 1. do **yo**u watch TV or videos with your child? | 🞎 | 🞎 | 🞎 | 🞎 | 🞎 | 🞎 |
| 1. do **you** send your child outside to play so you can do household chores in your home? | 🞎 | 🞎 | 🞎 | 🞎 | 🞎 | 🞎 |
| 1. do **you** take your child to the park to play? | 🞎 | 🞎 | 🞎 | 🞎 | 🞎 | 🞎 |

1. Place an "X" in the box below that best represents how often the following things happen during a normal week.

| **During a normal week, how often...** | **Never** | **Very rarely** | **Rarely** | **Some- times** | **Often** | **Very often** |
| --- | --- | --- | --- | --- | --- | --- |
| 1. do **you** tell your child that physical activity is good for health? | 🞎 | 🞎 | 🞎 | 🞎 | 🞎 | 🞎 |
| 1. does **your** behavior encourage your child to be sedentary? (*for example, watching TV for a long time.*) | 🞎 | 🞎 | 🞎 | 🞎 | 🞎 | 🞎 |
| 1. do **you** praise your child for participating in sports or physical activities? | 🞎 | 🞎 | 🞎 | 🞎 | 🞎 | 🞎 |
| 1. do **you** turn on the TV, a video, or movie for your child when the weather is bad? (*for example, when it is raining, too hot or too cold.)* | 🞎 | 🞎 | 🞎 | 🞎 | 🞎 | 🞎 |
| 1. do **you** say things to encourage your child to do physical activities or play sports? | 🞎 | 🞎 | 🞎 | 🞎 | 🞎 | 🞎 |

1. Place an "X" in the box below that best represents how often each of the following things happen during a normal week.

| **During a normal week, how often...** | **Never** | **Very rarely** | **Rarely** | **Some- times** | **Often** | **Very often** |
| --- | --- | --- | --- | --- | --- | --- |
| 1. does your child **hear you say** that you were too tired to do physical activities? | 🞎 | 🞎 | 🞎 | 🞎 | 🞎 | 🞎 |
| 1. does your child **see** **you** watching TV or movies? | 🞎 | 🞎 | 🞎 | 🞎 | 🞎 | 🞎 |
| 1. do **you** play sports, active games, or do other physical activities with your child? | 🞎 | 🞎 | 🞎 | 🞎 | 🞎 | 🞎 |
| 1. do **you** try to get your child to play outside when the weather is nice? | 🞎 | 🞎 | 🞎 | 🞎 | 🞎 | 🞎 |
| 1. do **you** transport your child to a place where they can be physically active or play sports? | 🞎 | 🞎 | 🞎 | 🞎 | 🞎 | 🞎 |

1. Place an "X" in the box below that best represents how often each of the following things happen during a normal week.

| **During a normal week, how often...** | **Never** | **Very rarely** | **Rarely** | **Some- times** | **Often** | **Very often** |
| --- | --- | --- | --- | --- | --- | --- |
| 1. does your child **hear you** talk about participating in a sport or being physically active? | 🞎 | 🞎 | 🞎 | 🞎 | 🞎 | 🞎 |
| 1. does your child **see you** doing, or going to do, something that is physically active (*for example, walking, biking, playing sports*)? | 🞎 | 🞎 | 🞎 | 🞎 | 🞎 | 🞎 |
| 1. do **you** turn on the TV, a video, or movie for your child so you can do household chores in your home? | 🞎 | 🞎 | 🞎 | 🞎 | 🞎 | 🞎 |
| 1. do **you** try to get your child to be physically active instead of watching TV? | 🞎 | 🞎 | 🞎 | 🞎 | 🞎 | 🞎 |
| 1. do **you** say things to encourage your child to spend less time being sedentary? (*for example, "Stop watching TV and go outside and play".)* | 🞎 | 🞎 | 🞎 | 🞎 | 🞎 | 🞎 |

1. Place an "X" in the box below that best represents how important each of the following statements is to you.

| **How important is it for you that your child…** | **Unimportant** | **Of little importance** | **moderately important** | **important** | **very important** |
| --- | --- | --- | --- | --- | --- |
| 1. participate in sports? | 🞎 | 🞎 | 🞎 | 🞎 | 🞎 |
| 1. be physically active when they grow up? | 🞎 | 🞎 | 🞎 | 🞎 | 🞎 |

| ***About your Interactions with your Child in Relation to Sleep*** |
| --- |

The following questions are about the ways you interact with your child in relation to sleep.

**To answer the questions remember that:**

Bedtime routines are a set sequence of activities that occur regularly in the same order and with the same caretaker before a child goes to bed. Place an "X" in the box that best represents your child's sleep routine in the last month.

| 1. **During *weeknights* for the past month, how often did your child…** | **Almost**  **never** | **Sometimes** | **Half the time** | **Often** | **Nearly**  **always** |
| --- | --- | --- | --- | --- | --- |
| 1. perform the **same activities** in the hour before going to bed *(for example, bathe, brush teeth, read/listen to story, listen to music)*? | 🞎 | 🞎 | 🞎 | 🞎 | 🞎 |
| 1. perform activities **in the same order** before going to bed *(for example, bathe, brush teeth, read/listen to story, listen to music)*? | 🞎 | 🞎 | 🞎 | 🞎 | 🞎 |
| 1. sleep **in the same place** *(for example, in his/her bed, in parent’s bed, on couch)*? | 🞎 | 🞎 | 🞎 | 🞎 | 🞎 |
| 1. go to bed **at the same time** (within 10 minutes of the scheduled time)? | 🞎 | 🞎 | 🞎 | 🞎 | 🞎 |
| 1. get put to bed **by the same person**? | 🞎 | 🞎 | 🞎 | 🞎 | 🞎 |

| 1. **During *weekend nights* for the past month, how often did your child…** | | **Almost**  **never** | | **Sometimes** | | | **Half the time** | | | | **Often** | | | | **Nearly**  **always** |  |  |
| --- | --- | --- | --- | --- | --- | --- | --- | --- | --- | --- | --- | --- | --- | --- | --- | --- | --- |
| 1. perform the **same activities** in the hour before going to bed *(for example, bathe, brush teeth, read/listen to story, listen to music)*? | | 🞎 | | 🞎 | | | 🞎 | | | | 🞎 | | | | 🞎 |  |  |
| 1. perform activities **in the same order** before going to bed *(for example, bathe, brush teeth, read/listen to story, listen to music)*? | | 🞎 | | 🞎 | | | 🞎 | | | | 🞎 | | | | 🞎 |  |  |
| 1. sleep **in the same place** *(for example, in his/her bed, in parent’s bed, on couch)*? | | 🞎 | | 🞎 | | | 🞎 | | | | 🞎 | | | | 🞎 |  |  |
| 1. go to bed **at the same time** (within 10 minutes of the scheduled time)? | | 🞎 | | 🞎 | | | 🞎 | | | | 🞎 | | | | 🞎 |  |  |
| 1. get put to bed **by the same person**? | | 🞎 | | 🞎 | | | 🞎 | | | | 🞎 | | | | 🞎 |  |  |
| 1. **How upset does your child get if he or she does NOT…** | **Not at all** | | **A little** | | **Moderately** | | | **Quite a bit** | | | | **Extremely** | | | |  |  |
| 1. perform the **same activities** in the hour before going to bed *(for example, bathe, brush teeth, read/listen to story, listen to music)*? | 🞎 | | 🞎 | | 🞎 | | | 🞎 | | | | 🞎 | | | |  |  |
| 1. perform activities **in the same order** before going to bed (*for example, bathe, brush teeth, read/listen to story, listen to music*)? | 🞎 | | 🞎 | | 🞎 | | | 🞎 | | | | 🞎 | | | |  |  |
| 1. sleep **in the same place** *(for example, in his/her bed, in parent’s bed, on couch*)? | 🞎 | | 🞎 | | 🞎 | | | 🞎 | | | | 🞎 | | | |  |  |
| 1. go to bed **at the same time** (within 10 minutes of the scheduled time)? | 🞎 | | 🞎 | | 🞎 | | | 🞎 | | | | 🞎 | | | |  |  |
| 1. get put to bed **by the same person**? | 🞎 | | 🞎 | | 🞎 | | | 🞎 | | | | 🞎 | | | |  |  |
|  | | | | | | | | | | | | | | | |  |  |
| 1. **In the past month, in the hour before going to bed, how often did your child…** | **Almost never** | | **Sometimes** | | | **Half the time** | | | | **Often** | | | | **Nearly always** | | | |
| 1. Read/listen to a story? | 🞎 | | 🞎 | | | 🞎 | | | | 🞎 | | | | 🞎 | | | |
| 1. Play with games or toys? | 🞎 | | 🞎 | | | 🞎 | | | | 🞎 | | | | 🞎 | | | |
| 1. Have active play (such as roughhouse or run around? | 🞎 | | 🞎 | | | 🞎 | | | | 🞎 | | | | 🞎 | | | |
| 1. Watch TV? | 🞎 | | 🞎 | | | 🞎 | | | | 🞎 | | | | 🞎 | | | |
| 1. Play video games? | 🞎 | | 🞎 | | | 🞎 | | | | 🞎 | | | | 🞎 | | | |
| 1. Listen to music? | 🞎 | | 🞎 | | | 🞎 | | | | 🞎 | | | | 🞎 | | | |
| 1. Have a snack or drink? | 🞎 | | 🞎 | | | 🞎 | | | | 🞎 | | | | 🞎 | | | |
| 1. Take a shower/bath? | 🞎 | | 🞎 | | | 🞎 | | | | 🞎 | | | | 🞎 | | | |
| 1. Brush teeth? | 🞎 | | 🞎 | | | 🞎 | | | | 🞎 | | | | 🞎 | | | |
| 1. Use the toilet? | 🞎 | | 🞎 | | | 🞎 | | | 🞎 | | | | 🞎 | | | |  |
| 1. Hug/kiss caregiver (*for example, a good night kiss*)? | 🞎 | | 🞎 | | | 🞎 | | | 🞎 | | | | 🞎 | | | |  |
| 1. Say goodnight to family members? | 🞎 | | 🞎 | | | 🞎 | | | 🞎 | | | | 🞎 | | | |  |
| 1. Get tucked in? | 🞎 | | 🞎 | | | 🞎 | | | 🞎 | | | | 🞎 | | | |  |
| 1. Put on pajamas? | 🞎 | | 🞎 | | | 🞎 | | | 🞎 | | | | 🞎 | | | |  |
| 1. Say prayers? | 🞎 | | 🞎 | | | 🞎 | | | 🞎 | | | | 🞎 | | | |  |
| 1. Cuddle with caregiver? (*for example, the child sat on the lap of the caregiver and was hugged with him/her)* | 🞎 | | 🞎 | | | 🞎 | | | 🞎 | | | | 🞎 | | | |  |

**You’re finished!**

**Thank you for your time and effort!**
