# Supplementary material for: Cross-Cultural Adaptation of Instruments Measuring Children’s Movement Behaviors and Parenting Practices in Brazilian Families
Source: Int J Environ Res Public Health. 2020 Dec 31;18(1):239. doi: 10.3390/ijerph18010239 (PMC7794996; doi:10.3390/ijerph18010239)
Supplement: Supplementary file 1 [file ijerph-18-00239-s001.zip › Supplementary File 4.docx]

**PARENTING PHYSICAL ACTIVITY PRACTICES QUESTIONNAIRE**

**Calculation of Scales: 6-factor Model around Controlling Practices**

- *Factor 1* = rules around active play indoors (higher score indicates more rules that restrict active play indoors)
  - Includes 13 items: ppa2.1A_Q.19, ppa2.1B+C_Q.19 (derived variable, combination of ppa2.1b and ppa2.1c), ppa2.1D_Q.19, ppa2.1E_Q.19, ppa2.1F_Q.19, ppa2.1G_Q.19, ppa2.1H_Q.19, ppa2.1I_Q.19, ppa2.1J_Q.19, ppa2.1K_Q.19, ppa2.2A__Q.20, ppa2.3B_R_Q.20, ppa2.4_Q.21
- *Factor 2* = rules around active play outdoors (higher score indicates greater rules around staying calm and clean during outside play)
  - Includes 3 items: ppa2.5A_Q.22, ppa2.6B_ppa2.10f_Q.22 (derived variable, average of ppa2.6 and ppa2.10), ppa2.9E_Q.22
- *Factor 3* = use of PA to reward/control child behavior (higher score indicates greater use of PA opportunities to reward/control child behavior)
  - Includes 5 items: ppa2.22C_Q.31, ppa2.23D_Q.31, ppa2.31B_Q.35, ppa2.33D_Q.35, ppa2.34E_Q.35
- *Factor 4* = limiting or monitoring of screen time (higher score indicates greater control over TV)
  - Includes 6 items: ppa2.13_R_Q.24, ppa2.15_R_Q.26, ppa2.17R_Q.28+19R_Q.30 (derived variable, average of ppa2.17 and ppa2.19, both items reverse scored), ppa2.24A+25B+26C+27D (derived variable, average of ppa2.24, ppa2.25, ppa2.26, and ppa2.27), ppa3.3A_Q.36, ppa3.22A_Q.47
- *Factor 5* = use of screen time to reward/control child behavior (higher score indicates greater use of screen time to reward/control child behavior)
  - Includes 4 items: ppa2.20A_Q.31, ppa2.21B_Q.31, ppa2.30A_Q.35, ppa2.32C_Q.35
- *Factor 6* = limiting outdoor play due to weather (higher score indicates greater rules restricting outdoor play)
  - Includes 2 items: ppa2.7C_R_Q.22, ppa2.8D_R_Q.22

**Notes:** R indicates item was reverse scored.

**PARENTING PHYSICAL ACTIVITY PRACTICES QUESTIONNAIRE**

**Calculation of Scales: 8-factor Model around Supportive Parenting Practices**

- *Factor 1* = explicit modeling and enjoyment of PA (higher scores indicates greater modeling and enjoyment of PA or lower modeling and enjoyment of sedentary by parent)
  - Includes 10 items: ppa3.6C_Q.36, ppa3.10_Q.37, ppa3.12_Q.39, ppa3.17_Q.44, ppa3.28D_Q.47, ppa4.11B_R_Q.49, ppa4.16A_R_Q.50, ppa4.18C_Q.50, ppa4.22A_Q.51, ppa4.23B_Q.51
- *Factor 2* = verbal encouragement for physical activity (higher scores indicate greater encouragement for physical activity)
  - Includes 7 items: ppa4.3A_Q.48, ppa4.5C_Q.48, ppa4.10A_Q.49, ppa4.12_Q.49, ppa4.14_Q.49, ppa4.25D_Q.51, ppa4.26E_Q.51
- *Factor 3* = logistic support for sports (higher scores indicates greater logistic support)
  - Includes 3 items: ppa3.13_Q.40, ppa3.16_CAT_Q.43 (derived variable, see categories below), ppa3.19_Q.45
- *Factor 4* = logistic support for active play (higher scores indicate greater logistic support)
  - Includes 4 items: ppa3.20_CAT_Q.46 (derived variable, see categories below), ppa4.6_Q.48, ppa4.19D_Q.50, ppa4.20E_Q.50
- *Factor 5* = importance and value of PA (higher scores indicate greater value for PA)
  - Includes 3 items: ppa3.14_Q.41, ppa4.27A_Q.52, ppa4.28B_Q.52
- *Factor 6* = support/reinforcement from other adults (higher score indicates greater support from other adults in household)
  - Includes 3 items: ppa3.4B_R_Q.36, ppa3.26B_Q.47, ppa3.27C_R_Q.47
- *Factor 7* = exposure to TV (higher scores indicates higher exposure to TV)
  - Includes 3 items: ppa1.6_Q.18, ppa2.28_Q.33, ppa2.29_Q.34
- *Factor 8* = explicit modeling and enjoyment of screen time (higher scores indicates greater modeling of screen time)
  - Includes 6 items: ppa3.7D_Q.36, ppa3.11_Q.38, ppa4.4B_Q.48, ppa4.13D_Q.49, ppa4.17B_Q.50, ppa4.24C_Q.51

**Notes:** R indicates item was reverse scored; CAT indicates item was categorized.

I - Derived variable: PPA3.16_CAT_Q.43

- Category 0 = 0 activities (PPA3.15_Q.42=0)
- Category 1 = 1 activity
- Category 2 = 2 activities
- Category 3 = 3 activities
- Category 4 = 4 activities
- Category 5 = 5 or more activities

II - Derived variable: PPA3.20_CAT_Q.46

- Category 0 = 0 trips in past month
- Category 1 = 1 trip in past month
- Category 2 = 2 trips in past month
- Category 3 = 3 trips in past month
- Category 4 = 4 trips in past month
- Category 5 = 5 trips in past month
- Category 6 = 6 trips in past month
- Category 7 = 7-8 trips in past month
- Category 8 = 9-10 trips in past month
- Category 9 = 11 or more trips in past month

III - Derived variable for PPA2.13_CAT_Q.24 (use as model for creating similar categories for PPA2.15_CAT_Q.26)

- Category 0 = 0 min/weekday
- Category 1 = 1-29 min/weekday
- Category 2 = 30-49 min/weekday
- Category 3 = 50-79 min/weekday
- Category 4 = 80-119 min/weekday
- Category 5 = 120-150 min/weekday
- Category 6 = 151-190 min/weekday
- Category 7 = 191 or more min/weekday
- Category 8 = no limits (PPA2.12_Q.23=0, or similar lead in like PPA2.14_Q.25)

**Categories are then reverse coded so that higher scores indicate more control over TV time.

IV - Derived variable for PPA2.17_CAT_Q.28 (use as model for creating similar categories for, PPA2.19_CAT_Q.30)

- Category 0 = 0 min/weekday
- Category 1 = 1-29 min/weekday
- Category 2 = 30-59 min/weekday
- Category 3 = 60-119 min/weekday
- Category 4 = 120 or more min/weekday
- Category 5 = no limits (PPA2.16_Q.27=0, or similar lead in like PPA2.18_Q.29)

**Categories are then reverse coded so that higher scores indicate more control over video games.

**BEDTIME ROUTINE QUESTIONNAIRE**

**Calculation of Scales: 5-factor Model around Bedtime Routine**

- *Factor 1* = consistency: routine behavior (higher score indicates greater routine behavior)
  - Includes 4 items: BRQ1.1A_Q.53, BRQ1.2B_Q.53, BRQ1.6A_Q.54, BRQ1.7B_Q.54
- *Factor 2* = consistency: routine environment (higher score indicates greater routine environment)
  - Includes 6 items: BRQ1.3C_Q.53, BRQ1.4D_Q.53, BRQ1.5E_Q.53, BRQ1.8C_Q.54, BRQ1.9D_Q.54, BRQ1.10E_Q.54
- *Factor 3* = reactivity (higher score indicates greater reactivity)
  - Includes 5 items: BRQ1.11A_Q.55, BRQ1.12B_Q.55, BRQ1.13C_Q.55, BRQ1.14D_Q.55, BRQ1.15E_Q.55
- *Factor 4* = adaptive activities (higher score indicates greater adaptive activities)
  - Includes 10 items: BRQ1.16A_Q.56, BRQ1.23H_Q.56, BRQ1.24I_Q.56, BRQ1.25J_Q.56, BRQ126K_Q.56, BRQ1.27L_Q.56, BRQ1.28M_Q.56, BRQ1.29N_Q.56, BRQ1,30*O*_Q.56, BRQ1.31P_Q.56
- *Factor 5* = maladaptive activities (higher score indicates maladaptive activities)
  - Includes 6 items: BRQ1.17B_Q.56, BRQ1.18C_Q.56, BRQ1.19D_Q.56, BRQ1.20E_Q.56, BRQ1.21F_Q.56, BRQ1.22G_Q.56
